# FDA first to global follow-on: alignment in expedited oncology approvals across EMA, TGA, and PMDA

**DOI:** 10.3389/fphar.2026.1804782

**Published:** 2026-05-29

**Authors:** Yehhyun Kim, Hisashi Urushihara, Gyeyoung Choi, Seungjin Bae

**Affiliations:** 1 College of Pharmacy, Ewha Womans University, Seoul, Republic of Korea; 2 Division of Drug Development and Regulatory Science, Faculty of Pharmacy, Keio University, Tokyo, Japan

**Keywords:** evidentiary maturity, expedited approval pathways, oncology approvals, regulatory comparison, regulatory concordance

## Abstract

**Introduction:**

Oncology drugs often receive their first expedited approval in the United States, yet the degree of alignment with subsequent regulatory decisions in other jurisdictions remains uncertain. We examined concordance in evidentiary evaluation and review timelines between the US Food and Drug Administration (FDA)-first expedited oncology approvals and subsequent decisions by the European Medicines Agency (EMA), Therapeutic Goods Administration (TGA), and Pharmaceuticals and Medical Devices Agency (PMDA).

**Methods:**

This study included all oncology drugs that received their first expedited approval from the FDA in 2019–2023. Subsequent EMA, TGA, and PMDA decisions were evaluated in pairwise comparison with the FDA. Concordance with the FDA was assessed for expedited pathway use, pivotal trial selection, and three analytical components (primary endpoint, target population, and data cut-off date (DCO)). Submission interval and review duration relative to the FDA were compared using Wilcoxon rank-sum tests.

**Results:**

Among 36 FDA expedited oncology approvals, EMA (n = 28), TGA (n = 18), and PMDA (n = 15) granted subsequent authorizations through expedited or standard pathways. TGA showed the highest concordance with the FDA, retaining expedited pathways in 72% of cases and closely mirroring the FDA’s analytical interpretations in the same pivotal trials. EMA maintained a similar rate of expedited use (71%) but often broadened target populations or used later DCOs (median +136 days). PMDA, in contrast, relied mainly on standard approvals (93%) with Japan-specific population analyses. Across all agencies, primary endpoint choice remained concordant with the FDA. EMA had the shortest submission lag (median 27 days), whereas TGA cases exceeded 600 days. All agencies had longer review duration than the FDA.

**Discussion:**

These findings highlight the need for international harmonization of regulatory frameworks and evidentiary thresholds to promote consistency in approval decisions and accelerate global access to innovative cancer therapies.

## 1 Introduction

Cancer remains a leading cause of morbidity and mortality worldwide, intensifying demand for timely access to new oncology treatments (National Cancer Institute, 2025). To address this, regulatory authorities have implemented expedited approval pathways that enable earlier market entry, contingent on later confirmatory trials (Mehta et al., 2022; Franco et al., 2023). The US Food and Drug Administration (FDA) was the first and most frequent adopter through its Accelerated Approval program (AA) launched in 1992 (Beakes-Read et al., 2022; Hwang et al., 2022; U.S. Food and Drug Administration, 2024), followed by comparable frameworks introduced by the European Medicines Agency (EMA; Conditional Marketing Authorization (CMA)) (European Medicines Agency, n.d.-a), the Australian Therapeutic Goods Administration (TGA; Provisional Approval) (Therapeutic Goods Administration, 2018), and the Japanese Pharmaceuticals and Medical Devices Agency (PMDA; Conditional Early Approval (CEA)) (Pharmaceuticals and Medical Devices Agency, 2017).

The global expansion of expedited approval programs has heightened debate regarding evidentiary robustness (Schnog et al., 2021; Sharma et al., 2025), as approvals often rely on single-arm trials with surrogate endpoints (Ribeiro et al., 2022; Huang et al., 2023; Ribeiro et al., 2023), while confirmatory studies take years to complete (Beaver et al., 2018; Tibau et al., 2025). The FDA typically serves as the first regulator to grant expedited approval, with other agencies subsequently reviewing nearly identical applications (Kuhler et al., 2019; Kashoki et al., 2020; Rohr et al., 2023). Nevertheless, multinational analyses indicate that the EMA, PMDA, and TGA grant expedited approvals far less frequently than the FDA (Hwang et al., 2022), reflecting divergent regulatory decisions across jurisdictions. The basis for such divergence, particularly how regulators evaluate identical applications, remains unclear.

To our knowledge, no prior study has systematically compared how different regulatory authorities interpret the same applications, despite their central role in shaping approval outcomes (Tafuri et al., 2014; Kuhler et al., 2019; Kashoki et al., 2020; Rohr et al., 2023). Previous studies have largely focused on differences in approval rates or expedited pathways—primarily between the FDA and EMA—rather than how the same clinical evidence is assessed under different regulatory standards within the same application. These approaches typically rely on classifications based solely on approval status or regulatory pathway. In contrast, our study introduces a six-level concordance framework to capture trial-level differences in the application of regulatory standards, extends the analysis to include the TGA and PMDA for a more globally representative comparison, and links these differences to submission and review timelines to incorporate a temporal dimension.

This study assessed cross-agency concordance in expedited oncology approvals, focusing on both evidentiary evaluation and review timing. Specifically, we identified three key trial-level dimensions that capture sources of evidentiary divergence. First, *who* is treated*:* the definition of the target population determines patient eligibility, thus if regulators defined the same trial population differently, this can yield divergent efficacy judgments (Sumi et al., 2020; Schnog et al., 2021; Perini et al., 2025). Second, *what* counts as benefit: the choice of primary endpoint sets the evidentiary standard, with agencies differing in their reliance on surrogate measures (Salcher-Konrad et al., 2020; Xie et al., 2023). Third, *how mature* the evidence is: the data cut-off date (DCO) reflects the follow-up completeness (Croft, 2018; Patel, 2024), meaning agencies may base their decisions on analyses of varying maturity. This dimension is further shaped by submission lags and differences in review duration, as these temporal gaps can lead regulators to evaluate the same trial at different stages of evidence accumulation (Tafuri et al., 2014; Hwang et al., 2020; Lythgoe et al., 2022).

Using the FDA-first expedited oncology approvals from 2019 to 2023 as the benchmark, we compared subsequent EMA, TGA, and PMDA decisions to identify where and how divergence emerges.

## 2 Methods

### 2.1 Overview of study design

This cross-sectional study examined divergence in approval pathways, evidentiary evaluations, and submission/review timelines following FDA-first expedited approvals of oncology drug–indication pairs. Subsequent regulatory decisions by the EMA, TGA, and PMDA were analyzed, as these agencies—alongside the FDA—exert significant influence over the global oncology market (Centre for Innovation in Regulatory Science CIRS, 2024). These authorities publish publicly comparable assessment reports and operate expedited pathways parallel to the FDA’s Accelerated Approval—namely Conditional Marketing Authorization (EMA), Provisional Approval (TGA), and Conditional Early Approval (PMDA) (Mehta et al., 2022; Franco et al., 2023) (Supplementary Table 1). Consistent with prior comparative analyses (Mehta et al., 2022; Vokinger et al., 2022; Franco et al., 2023; Xie et al., 2023), we distinguished expedited *approvals*—which grant temporary marketing authorization based on preliminary efficacy evidence—from expedited *review* programs primarily designed to facilitate drug development or shorten regulatory review timelines such as FDA Fast Track, Priority Review, or Breakthrough Therapy Designation; EMA Accelerated Assessment; TGA Priority Review; and PMDA SAKIGAKE (Franco et al., 2023; Centre for Innovation in Regulatory Science CIRS, 2024). These review programs were therefore excluded from direct FDA–subsequent agency approval comparisons.

The index cohort comprised oncology indications receiving the FDA’s expedited approval between 1 Jan 2019, and 31 Dec 2023, with subsequent EMA, TGA, and PMDA decisions tracked through 31 Jan 2025. The study period was selected to reflect the point at which all expedited approval pathways were in effect, with the TGA’s Provisional Approval being the last to be implemented in 2018 (Therapeutic Goods Administration, 2018).

### 2.2 Data extraction

#### 2.2.1 Identification of FDA-First expedited oncology approvals

To identify FDA-first expedited oncology approvals, we reviewed the FDA’s annual *Novel Drug Approvals* reports from 2019 to 2023 (U.S. Food and Drug Administration, 2023). These reports specify expedited approval status and flag products designated as “First in the United States”. The unit of analysis was the drug–indication pair, treating each indication as a distinct evaluative event (Salcher-Konrad et al., 2020; Lythgoe et al., 2022; Liu et al., 2024). Each entry was standardized by International Nonproprietary Name (INN) and categorized by indication based on the World Health Organization (WHO) Anatomical Therapeutic Chemical (ATC) classification. We included only new molecular entities indicated for solid or hematologic malignancies. We excluded supplementary indications of previously approved drugs, supportive therapies, biosimilars, and new formulations (Lythgoe et al., 2022). Gene and cell therapies were also excluded due to their distinct regulatory frameworks—such as the FDA’s Regenerative Medicine Advanced Therapy Designation—which could introduce heterogeneity in evidentiary standards (Drago et al., 2021; Yoon et al., 2024).

#### 2.2.2 Identification of subsequent regulatory actions by the EMA, TGA, and PMDA

For each FDA-first oncology expedited approval, subsequent regulatory decisions by the EMA, TGA, and PMDA were identified through searches of the following official databases: the EMA Medicines database (https://www.ema.europa.eu/en/medicines), the Australian Register of Therapeutic Goods (ARTG; https://www.tga.gov.au/resources/artg), and the PMDA Approved Drug Information database (https://www.pmda.go.jp/english/review-services/reviews/approved-information/drugs/0003.html). The searches were conducted between 9 June 2025, and 30 September 2025. For each drug–indication pair, approval records and official regulatory review reports were searched and retrieved directly from these publicly accessible sources using the International Nonproprietary Name (INN) as the search term.

Cases where the subsequent agency granted either expedited or standard marketing authorization were classified as “subsequently approved,” regardless of later withdrawal. Only the first approval decision per agency was included in the analysis; post-approval modifications—including withdrawals, indication expansions or restrictions, and re-examination outcomes—were excluded. Cases where no approval record was identified in the agency’s database or official documents were classified as “not approved.” For each subsequently approved case, the date of first approval was recorded and linked to the corresponding official regulatory review report published by each agency. These reports served as the primary source for the extraction of procedural and evidentiary variables described in Section 2.2.3.

#### 2.2.3 Extraction of procedural and evidentiary variables

For all cases with recorded approval, we extracted structured data on procedural and evidentiary domains from publicly available regulatory documents from the FDA (U.S. Food and Drug Administration, n.d.), EMA (European Medicines Agency, n.d.-b), TGA (Therapeutic Goods Administration, 2018), and PMDA (Pharmaceuticals and Medical Devices Agency, n.d.). Procedural variables included the approval pathway (expedited or standard) and key regulatory milestones (Zosso-Pavic et al., 2024) (submission and approval dates). For the EMA, the EC decision date was used as the approval date; for the TGA, the ARTG registration date was used.

Evidentiary variables captured pivotal trial characteristics and analytical components of efficacy evaluation. A pivotal trial was defined as the clinical study (or studies) formally cited by the regulatory agency as the primary evidentiary basis for approval in its official assessment report; in cases where multiple trials were cited, all were included in the analysis (Downing et al., 2014). Pivotal trial(s) were identified using *ClinicalTrials.gov* National Clinical Trial (NCT) identifiers, and key design features—randomization, masking, and phase—were recorded. Subsequently, three analytical components were extracted: target population, primary endpoint, and DCO date (2010). The target population referred to the cohort forming the evidentiary basis for approval (e.g., intention-to-treat (ITT), modified intention-to-treat (mITT), or per-protocol (PP) population). When sponsors submitted multiple population analyses, the population selected by the regulatory agency as the evidentiary basis for approval was used in this study. As this selection process is embedded within regulatory review and not systematically reported as a discrete discrepancy, differences between sponsor-submitted and regulator-selected populations could not be independently identified or quantified. Primary endpoints were based on the final determination in agency reviews (e.g., overall survival (OS), progression-free survival (PFS), overall response rate (ORR)). When multiple updates were available, the most recent regulator-accepted DCO date was recorded.

### 2.3 Assessment of concordance

#### 2.3.1 Definitions of concordance

We assessed concordance across two domains: *procedural and evidentiary*. *Procedural* concordance reflected whether the subsequent agency used the same approval pathway as the FDA (expedited vs. standard). Subsequent expedited approvals in alignment with the FDA were considered concordant. *Evidentiary* concordance was evaluated by first determining whether the pivotal trial(s) supporting subsequent approvals were identical to those referenced by the FDA. When identical, three analytical components—target population, primary endpoint, and DCO date—were compared. Each component was classified as concordant when consistent with the FDA’s assessment. *Analytical divergence* was defined as any discordance in at least one of these three components among approvals based on the same pivotal trial(s) as the FDA. Detailed definitions are provided in the *Supplementary Methods*. Non-approvals and approvals lacking publicly available review reports were excluded from concordance analysis.

#### 2.3.2 Classification of concordance

**FIGURE 1:**
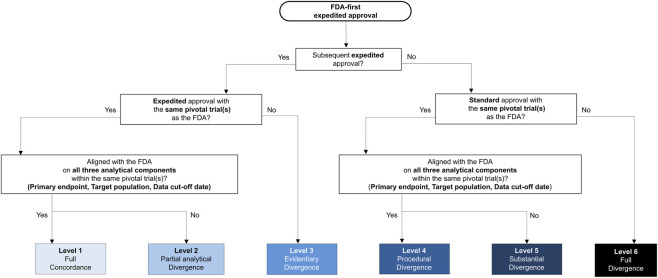
Six-level concordance framework for subsequent regulatory decisions relative to FDA-first expedited approvals. This framework categorizes the degree of concordance between the FDA-first expedited approvals and subsequent agencies’ approval decisions. The concordance classification proceeds sequentially from the procedural to the evidentiary domain. Procedural concordance was determined by comparing the type of approval pathway (expedited vs. standard). Evidentiary concordance was assessed based on whether agencies relied on the same pivotal trial(s) as the FDA and showed agreement in three analytical components: primary endpoint, target population, and data cut-off date (DCO). The framework ranges from Level 1 (full concordance) to Level 6 (full divergence), reflecting increasing regulatory misalignment. See Supplementary methods for further details on analytical concordance criteria. Abbreviations: FDA, US Food and Drug Administration.

We developed a six-level classification framework to assess the degree of concordance between FDA-first expedited approvals and subsequent regulatory decisions by the EMA, TGA, and PMDA (Figure 1). This framework incorporates both procedural and evidentiary dimensions, categorizing subsequent expedited approvals as levels 1–3, and standard approvals as levels 4–6. For expedited approvals, Level 1 (Full Concordance) was assigned when the subsequent agency relied on the same pivotal trial(s) as the FDA and was fully aligned across all three analytical components: target population, primary endpoint, and DCO date. Level 2 (Partial Analytical Divergence) was assigned when the same pivotal trial(s) were used but at least one analytical component differed, and level 3 (Evidentiary Divergence) when different pivotal trial(s) formed the basis of approval. Levels 4–6 reflected the same gradation among standard approvals, ranging from procedural divergence to substantial or full evidentiary divergence. Examples for each level are provided in Supplementary Tables 3, 5.

### 2.4 Outcome measures and statistical analysis

For cases sharing the same pivotal trial(s) as the FDA (Levels 1–2 and 4–5), component-wise analytical concordance was quantified. For DCO, intervals were calculated as the number of days between the subsequent agency’s DCO and the FDA’s, with positive values indicating later DCO use by the subsequent agency. Submission and review timelines were compared between expedited (Levels 1–3) and standard (Levels 4–6) approvals. Submission gaps (days between FDA and subsequent agency submissions) and review duration (days from submission to approval) were measured for each agency. Review duration includes both agency assessment time and any clock-stop periods during which the agency awaits sponsor responses.

Analyses were limited to drug-indication pairs approved by both the FDA and each subsequent agency. Categorical variables and concordance distributions were compared using the Fisher–Freeman–Halton exact test (Freeman and Halton, 1951; Agresti, 1992), with Pairwise Fisher’s exact tests for *post hoc* analyses, and Holm-Bonferroni correction for multiple comparisons (Holm, 1979; Lee and Lee, 2018). For continuous variables (e.g., DCO interval, submission gap, and review duration) normality was assessed using the Shapiro–Wilk test; if violated, the Wilcoxon rank-sum test was applied. Medians and interquartile ranges (IQRs) were reported for all temporal metrics. All analyses were conducted using Microsoft Excel and R software (version 4.4.1), with a two-sided *P* value of <0.05 considered statistically significant.

## 3 Results

### 3.1 Overview of FDA-first expedited oncology approvals and subsequent EMA, TGA, and PMDA approvals

**FIGURE 2:**
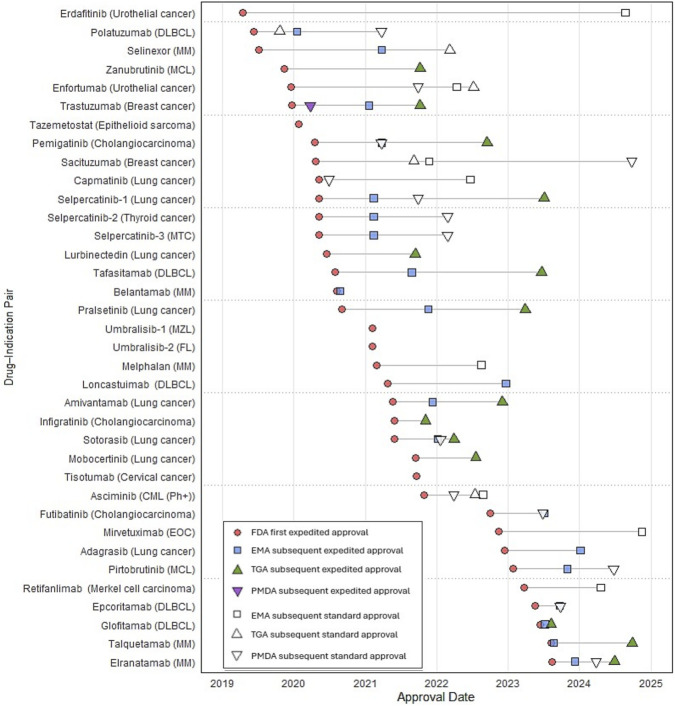
FDA-first expedited oncology approvals and subsequent regulatory decisions by EMA, TGA, and PMDA (2019–2023). Each symbol represents the approval date by a regulatory agency, shaped and colored according to the agency and approval pathway (expedited vs. standard). Horizontal lines indicate the time elapsed between the FDA-first expedited approval and subsequent approvals by other regulators. Drug–indication pairs not subsequently approved by a given agency are not shown. Two TGA’s subsequent expedited approvals (Epcoritamab, Talquetamab) were included based on publicly disclosed outcomes, despite the absence of review documents. When approval dates coincide or fall within the same month, overlapping symbols may represent more than one approval; exact approval dates and complete regulatory status for all drug–indication pairs are provided in Supplementary Table 2. Abbreviations: CML = chronic myeloid leukemia; DLBCL = diffuse large B-cell lymphoma; EMA = European Medicines Agency; EOC = epithelial ovarian cancer; FDA = US Food and Drug Administration; FL = follicular lymphoma; MCL = mantle cell lymphoma; MM = multiple myeloma; MTC = medullary thyroid cancer; MZL = marginal zone lymphoma; PMDA = Pharmaceuticals and Medical Devices Agency; TGA = Therapeutic Goods Administration.

Between 1 January 2019 and 31 December 2023, the FDA granted expedited approval for 36 oncology drug–indication pairs, preceding all other agencies (Supplementary Table 2). Figure 2 illustrates the chronological sequence of subsequent approvals by the EMA (n = 28), TGA (n = 20), and PMDA (n = 15), aligned with the date of FDA approval. Beyond timing differences, Figure 2 highlights marked variations in the use of expedited pathways across agencies. The EMA subsequently approved 28 of the FDA-led pairs, mostly via expedited pathways (20/28), while 8 proceeded through the standard pathways. The TGA showed a similar pattern, approving 20 pairs, including 15 expedited and 5 standard approvals. In contrast, the PMDA approved only 15 of the pairs, relying almost exclusively on the standard pathway (14/15), with expedited status granted in just one case.

Pivotal evidence accepted by the EMA (n = 28), TGA (n = 19), and PMDA (n = 18) was broadly consistent with that underpinning FDA first-expedited approvals (n = 39) (Table 1). All pivotal trials were open-label, predominantly single-arm in designs (FDA 87%, EMA 75%, TGA 79%, PMDA 78%; p = 0·58) and based largely on early-phase evidence (FDA 95%, >80% for other agencies; p = 0·64). Most trials involved small patient populations, with ≤200 participants in 97% of FDA and 83% of PMDA trials, compared with 79% in the EMA and TGA (p = 0·04). Although the overall difference in target population size across agencies was statistically significant, no pairwise comparison reached significance (Supplementary Table 3). Surrogate endpoints predominated across all agencies (FDA 100%, EMA 93%, TGA 95%, PMDA 94%; p = 0·31), with minimal reliance on OS.

**TABLE 1:** Characteristics of pivotal trials supporting FDA-first expedited oncology approvals and subsequent EMA, TGA, and PMDA approvals.

Characteristics	Pivotal trials^a^, No. (%)				p-value^d^
	FDA	EMA	TGA	PMDA	
Total	39 (100)	28 (100)	19 (100)	18 (100)	​
Masking	​	​	​	​	NA
Open	39 (100)	28 (100)	19 (100)	18 (100)	​
Double-blind	0 (0)	0 (0)	0 (0)	0 (0)	​
Randomization	​	​	​	​	0·58
Double-arm RCT	5 (13)	7 (25)	4 (21)	4 (22)	​
Single-arm non-RCT	34 (87)	21 (75)	15 (79)	14 (78)	​
Phase^b^	​	​	​	​	0·64
1	2 (5)	1 (4)	1 (5)	0 (0)	​
1/2	8 (21)	7 (25)	5 (26)	2 (11)	​
2	27 (69)	15 (54)	10 (53)	13 (72)	​
3	2 (5)	5 (18)	3 (16)	3 (17)	​
Target population size^c^	​	​	​	​	0·04
<200	38 (97)	22 (79)	15 (79)	15 (83)	​
≥200	1 (3)	6 (21)	4 (21)	3 (17)	​
Primary endpoint	​	​	​	​	0·31
OS	0 (0)	2 (7)	1 (5)	1 (6)	​
Surrogate endpoint	39 (100)	26 (93)	18 (95)	17 (94)	​
PFS	0 (0)	2 (7)	1 (5)	1 (6)	​
ORR	37 (95)	21 (75)	14 (74)	13 (72)	​
CR	1 (3)	2 (7)	2 (11)	2 (11)	​
MMR	1 (3)	1 (4)	1 (5)	1 (6)	​

This table summarizes the characteristics of pivotal trials supporting oncology drug–indication pairs first approved through expedited approval pathways by the FDA (n = 36) between 2019 and 2023, together with subsequent approvals by the EMA (n = 28), TGA (n = 18), and PMDA (n = 15). Subsequent approvals by the EMA, TGA, and PMDA, included both expedited and standard pathways, according to each agency’s classification. Two TGA’s subsequent expedited approvals (Epcoritamab, Talquetamab) were excluded from the analysis owing to the absence of publicly available review documents.

^a^ The unit of analysis is the pivotal trial that served as the basis for each drug–indication pair’s approval. When a single drug–indication pair was supported by multiple pivotal trials, each trial was counted separately. Therefore, the number of pivotal trials may differ from the number of approved drug–indication pairs.

^b^ Trial phase classification follows regulatory documentation. Trials designated as “Phase 1/2” indicate combined designs with both Phase 1 and Phase 2 elements. If approval was based solely on Phase 2 data from such trials, they were classified as Phase 2.

^c^ Target population size refers to the number of patients forming the basis for efficacy evaluation and the scope of the approved indication.

^d^ *p*-values for overall comparisons across the FDA, EMA, TGA, and PMDA, were calculated using the Fisher–Freeman–Halton exact test and are presented in the rightmost column for each variable. Pairwise Fisher exact test p-values comparing agencies are presented separately in Supplementary Table 3.

Abbreviations: FDA = US, food and drug administration; EMA, european medicines agency; TGA, therapeutic goods administration; PMDA, pharmaceuticals and medical devices agency; RCT, randomized controlled trial; OS, overall survival; PFS, progression-free survival; ORR, objective response rate; CR, complete response; MMR, major molecular response; NA, not applicable.

### 3.2 Classification of subsequent approvals by three agencies based on procedural and evidentiary concordance with the FDA

Among subsequent regulatory approvals by the EMA (n = 28), TGA (n = 20), and PMDA (n = 15), two TGA cases were excluded due to unavailable review documents, leaving 28 EM A, 18 TG A, and 15 PMDA approvals for evaluation using the 6-level concordance framework (Table 2; Supplementary Table 4). Concordance levels differed significantly across agencies (p < 0·0001), reflecting variation in pathway designation, pivotal trial selection, or interpretation of three analytical components. TGA showed a substantial proportion of full concordance (Level 1), with 28% (5/18) of cases fully aligning with the FDA. However, these Level 1 cases did not demonstrate shorter review durations compared with other TGA approvals (median 394 vs. 382 days; p = 0.588). Most EMA decisions (68%, 19/28) were Level 2, based on the same pivotal trial(s) but diverging in at least one analytical component. PMDA exhibited the lowest concordance, with 60% (9/15) cases at Level 5, using the same pivotal trial(s) but applying a standard pathway. One case (7%, 1/15) in the PMDA was classified as Level 4—standard approval despite evidentiary alignment—whereas no Level 3 cases were observed across the three agencies. Level 6 cases in the EMA (14%, 4/28) and TGA (11%, 2/18) were based on confirmatory trials that supported FDA conversion to standard approval, whereas PMDA cases (27%, 4/15) additionally involved Japan-specific bridging trials (Supplementary Table 5).

**TABLE 2:** Classification of subsequent approvals by EMA (n = 28), TGA (n = 18), and PMDA (n = 15) based on regulatory concordance with FDA-first expedited approvals (Levels 1–6).

Concordance level^a^	Drug-indication pairs, no. (%)			p-value^b^
	EMA	TGA	PMDA	
Total	28 (100)	18 (100)	15 (100)	<0·0001
Level 1 full concordance	1 (4)	5 (28)	0 (0)	
Level 2 partial analytical divergence	19 (68)	8 (44)	1 (7)	
Level 3 evidentiary divergence	0 (0)	0 (0)	0 (0)	
Level 4 procedural divergence	0 (0)	0 (0)	1 (7)	
Level 5 substantial divergence	4 (14)	3 (17)	9 (60)	
Level 6 full divergence	4 (14)	2 (11)	4 (27)	

Values are presented as n (%), percentages indicate the proportion of each agency’s total subsequent approvals of FDA-led, cases. Two TGA, cases were excluded from the classification due to the lack of publicly available regulatory review documents, which prevented the assessment of pivotal trial and analytical concordance. Sums may not total to 100% because of rounding.

^a^ Concordance levels were defined to reflect increasing degrees of divergence from the FDA-first expedited approval across the three agencies (EMA, TGA, and PMDA). Level 1 indicates full concordance, defined as an expedited approval based on the same pivotal trial(s) with alignment across all three analytical components (primary endpoint, target population, and data cut-off date). Level 2 represents partial analytical divergence, where the subsequent agency granted an expedited approval based on the same pivotal trial(s) but differed in at least one analytical component. Level 3 reflects evidentiary divergence with an expedited approval based on different pivotal trial(s). Level 4 captures procedural divergence, defined as a standard approval based on the same pivotal trial(s), with analytical concordance. Level 5 indicates substantial divergence, where the agency granted a standard approval based on the same pivotal trial(s) with at least one analytical difference. Level 6 represents full divergence, where the agency issued standard approval based on different pivotal trial(s) with no analytical concordance.

^b^ The p-value was derived using Fisher–Freeman–Halton exact test, comparing the overall distribution of regulatory concordance levels (Levels 1–6) across the three agencies (EMA, TGA, and PMDA). This test evaluates whether the frequencies of all six concordance levels differ significantly between agencies. Pairwise p-values comparing agencies were obtained using Fisher’s exact test and Holm-Bonferroni correction, and are presented separately in Supplementary Table 4.

Abbreviations: EMA, european medicines agency; TGA, therapeutic goods administration; PMDA, pharmaceuticals and medical devices agency.

### 3.3 Analytical divergence among approvals based on the same pivotal trial

**FIGURE 3:**
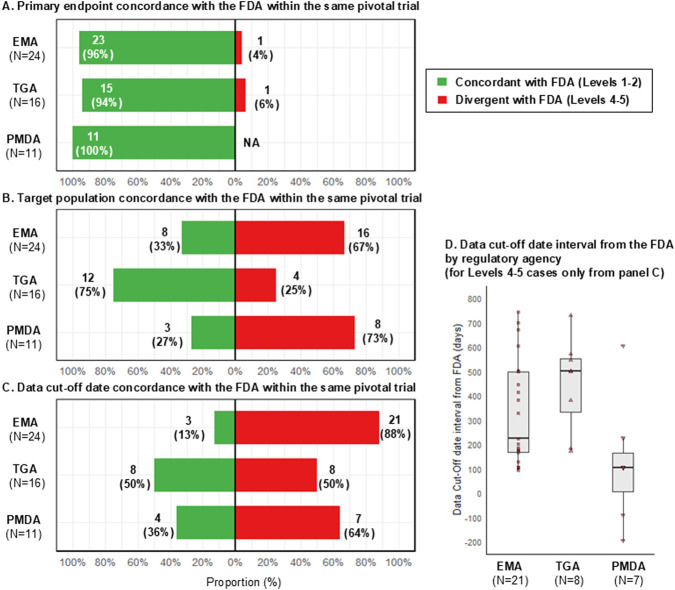
Comparison of three analytical components—target population, primary endpoint, and data cut-off date—across agencies for subsequent approvals based on the same pivotal trial as the FDA-first expedited approval. Comparisons were restricted to drug–indication pairs that received first expedited approval from the FDA and were subsequently approved using the same pivotal trial(s) by the EMA, TGA, or PMDA (N shown per agency). Analyses included only approvals classified as Concordance Levels 1–2 or 4–5, according to the regulatory concordance framework. Bar charts show the proportion of drug–indication pairs classified as concordant with the FDA (green) or divergent from the FDA (red) across three analytical variables: **(A)** primary endpoint, **(B)** target population, and **(C)** data cut-off date. For each bar, the n (%) outside the bar indicates the total for concordant or divergent cases, Percentages may not total 100% due to rounding. For panel **(C)**, boxplot panel **(D)** is additionally provided to show the distribution of DCO date intervals (days) in divergent cases relative to the FDA for each agency, displaying medians and interquartile ranges (IQR). Analytical concordance was assessed as follows: **(A)** Primary endpoint—the main efficacy outcome specified in each agency’s regulatory review; **(B)** Target population—the patient cohort forming the basis for regulatory approval and the scope of the approved indication; **(C)** Data cut-off date—the latest point of data maturity used in the efficacy analysis. A component was considered concordant if the subsequent agency used the same analytic specification as the FDA, and divergent if a different endpoint, population, or data cut-off date was applied. Abbreviations: FDA, US Food and Drug Administration; EMA, European Medicines Agency; TGA, Therapeutic Goods Administration; PMDA, Pharmaceuticals and Medical Devices Agency; NA, Not Applicable.

For subsequent approvals based on the same pivotal trial(s) as the FDA, analytical concordance was assessed for Levels 1–2 and 4–5, involving 24 EMA, 16 TGA, and 11 PMDA approvals (Figure 3; Supplementary Table 6). For the target population, the PMDA showed greatest divergence, with 73% (8/11) differing from the FDA, due to analyses restricted to Japan. The EMA diverged in 67% (16/24) of cases, including 11 approvals with broader populations than the FDA, whereas the TGA showed higher alignment, diverging in only 25% (4/16) of cases. Regarding the DCO dates, the EMA diverged in 88% (21/24) of cases, typically using later DCO dates (median extension 193 days; IQR 105–458), which were longer for standard than expedited approvals (median 586 vs. 176 days; p = 0·015) (Supplementary Table 7). The PMDA diverged in 64% (7/11) of cases with a median extension of 0 days (IQR 0–105), due to multiple cases with identical DCOs and a smaller number with later cut-off dates. The TGA diverged in 50% (8/16), with a median extension of 87 days (IQR 0–500). Nevertheless, primary endpoints remained highly consistent across all three agencies relative to the FDA.

### 3.4 Submission gap and review duration

Submission and review timelines varied by agency and by procedural concordance (Table 3) The EMA showed minimal submission delays for Levels 1–3 (median, 27 days; IQR 14.5–96.5) but substantial lags for Levels 4–6 (median, 521 days; IQR 268–734; p = 0·0032). Review duration was consistently longer than the FDA’s for both strata—419 vs. 214 days for Levels 1–3 (p < 0·0001) and 414 vs. 216 days for Levels 4–6 (p = 0·014). The TGA exhibited prolonged submission lags—324 days (IQR 157–679) for Levels 1–3 and 626 days (IQR 117–849) for Levels 4–6—and longer review duration than the FDA for Level 1–3 (394 vs. 182 days; P < 0·0001), with no significant difference for Levels 4–6 (322 vs. 173 days; p = 0·40). PMDA submissions were mainly Levels 4–6 with extended submission intervals (367 days; IQR 144–593); the only Level 1–3 case was filed 11 days after the FDA. Review duration exceeded the FDA’s in both strata (Level 1–3: 198 vs. 113 days, p > 0·99; Levels 4–6: 252 vs. 168 days, p = 0·080), although neither comparison reached statistical significance.

**TABLE 3:** Comparison of submission gap and review duration for oncology drug–indication pairs that received first expedited approval from the FDA and were subsequently granted either expedited (concordance levels 1–3) or standard approval (levels 4–6) by the EMA, TGA, and PMDA.

Measure	​	FDA	EMA	p-value	​	FDA	TGA	p-value	​	FDA	PMDA	p-value
	N	Median (IQR)	Median (IQR)		N	Median (IQR)	Median (IQR)	​	N	Median (IQR)	Median (IQR)	
Submission gap, days												
Overall	28	NA	45 (15·8–272)	NA	18	NA	362 (127–718)	NA	15	NA	350 (112–581.5)	NA
Concordance level												
1–3	20	NA	27 (14·5–96·5)	NA	13	NA	324 (157–679)	NA	1	NA	11 (11–11)	NA
4–6	8	NA	521 (268–734)	NA	5	NA	626 (117–849)	NA	14	NA	367 (144–593)	NA
Review duration, days												
Overall	28	214 (161–241)	419 (351–472)	<0·0001	18	180 (158–223)	388 (337–420)	<0·0001	15	163 (156–239)	238 (199–274)	0·053
Concordance level												
1–3	20	214 (164–242)	419 (351–485)	<0·0001	13	182 (163–214)	394 (349–413)	<0·0001	1	113 (113–113)	198 (198–198)	>0·99
4–6	8	216 (154–234)	414 (375–430)	0·014	5	173 (156–331)	322 (194–461)	0·40	14	252 (200–276)	252 (200–276)	0·080

This table presents two procedural timelines for oncology drug–indication pairs first approved by the FDA, through expedited pathways and subsequently approved by the EMA, TGA, or PMDA, stratified by regulatory concordance level. Submission gap refers to the interval (days) between the FDA’s original submission and the sponsor’s subsequent submission to each agency. Review duration refers to the interval (days) from the submission date to the approval date for each agency, with matched FDA, review time provided for reference.

Concordance Levels 1–3 represent subsequent expedited approvals, and Levels 4–6 represent subsequent standard approvals. The “Overall” row aggregates all follow-up cases per agency regardless of concordance level. Data are reported as median values with interquartile ranges (IQR). p-values were calculated using the Wilcoxon rank-sum test for paired comparisons.

Abbreviation: IQR, interquartile range; FDA = US, food and drug administration; EMA, european medicines agency; TGA, therapeutic goods administration; PMDA, pharmaceuticals and medical devices agency; NA, not applicable.

## 4 Discussion

This study provides the first systematic, multi-agency evaluation of expedited oncology approvals across the FDA, EMA, TGA, and PMDA, revealing significant procedural and evidentiary divergence despite shared goals of accelerating patient access. Using FDA-first expedited approvals as the reference, we identified heterogeneity in pathway designation, pivotal trial interpretation, and submission/review timelines.

The EMA showed high procedural concordance with the FDA but differed in evidentiary requirements within its regulatory framework. When reviewing the same trials, EMA often required later data cut-offs, which may reflect its emphasis on evidence maturity (Tafuri et al., 2014; European Medicines Agency, 2019; Kashoki et al., 2020). Notably, our observation of broader population definitions contrasts with other findings suggesting that the EMA tends to restrict indications relative to the FDA (Perini et al., 2025), while others found no major difference (Trotta et al., 2011; Rohr et al., 2023). In expedited contexts, where pivotal trials analyze 200 or fewer patients, these observed features—namely the use of later data cut-offs and broader population definitions—may reflect the EMA’s regulatory emphasis on ensuring sufficient evidence maturity and population characterization within the constraints of conditional approval. In contrast, the PMDA showed low procedural and evidentiary concordance, favoring standard pathways and relying more on domestic data. This finding aligns with critiques that Japan’s conditional approval functions more like a full approval, limiting regulatory flexibility (Miyazaki et al., 2022; Tanaka et al., 2023; Pharmaceutical Safety and Environmental Health Bureau Ministry of Health, 2024). Importantly, such divergence may partly reflect structural differences in eligibility criteria. For instance, the PMDA’s CEA is restricted to specific contexts (e.g., orphan), potentially limiting procedural concordance independent of differences in regulatory standards. Analyses of submission and review timelines further highlighted this divergence; lower-concordance cases (Levels 4–6) involving the EMA and PMDA were associated with delayed submissions, which may reflect stricter evidentiary standards or sponsor decisions to await confirmatory data before initiating submissions in other markets. Even when submission timing was similar, the EMA’s review durations remained longer than the FDA’s, suggesting that interpretive differences—such as population scope or data maturity—may extend internal assessment irrespective of procedural alignment.

In addition, the FDA’s acceptance of surrogate or biomarker-based endpoints may indicate a higher tolerance for evidentiary uncertainty, potentially contributing to earlier approvals and cross-agency differences in regulatory standards.

The TGA exhibited relatively high procedural and evidentiary concordance with the FDA but not consistently achieve faster approvals. Compared with the EMA, TGA submissions faced greater delays, likely due to resource constraints, sequential submission strategies, and differing market priorities (Zeukeng et al., 2018). This pattern is also consistent with the TGA’s Comparable Overseas Regulators (COR) framework, under which decisions by recognized agencies can serve as the primary evidentiary basis for TGA assessment. The five Level 1 concordance cases in our TGA dataset, in which full analytical alignment with the FDA was observed, are consistent with direct reliance on FDA review documentation. Accordingly, the longer submission gaps observed for the TGA may be associated with a sequential submission strategy by design rather than regulatory inefficiency: sponsors await FDA authorization before initiating TGA submission, after which the TGA conducts a targeted assessment leveraging the existing FDA review.

Overall, while all agencies share the goal of expediting access to promising cancer therapies, convergence in regulatory practice remains limited. Pivotal trials supporting expedited approvals are predominantly non-randomized, single-arm studies enrolling small populations and relying on surrogate endpoints. These evidentiary constraints may contribute to divergent regulatory interpretations—particularly in defining target populations and cut-off dates—and procedural gaps in submission and review duration. Prior studies indicate that patients and clinicians value durable clinical benefit and robust evidence over rapid access (Forrest et al., 2024; Koole et al., 2024). This societal expectation for evidentiary certainty underscores that the current divergence in how agencies define acceptable evidence for expedited approval may create inconsistent standards for what constitutes sufficient proof of benefit across jurisdictions.

Harmonizing evidentiary standards could therefore advance both timely access and global consistency. Key priorities include validating surrogate endpoints (Knottnerus and Knottnerus, 2022; Manyara et al., 2023), incorporation of longer follow-up with interim reassessment (Benjamin and Lythgoe, 2023), and earlier initiation of confirmatory trials in parallel with conditional approvals (Akhade et al., 2022). Broader and earlier cross-agency collaboration—through initiatives such as Project Orbis (de Claro et al., 2020)—may further facilitate coordinated submissions and concurrent reviews (Zosso-Pavic et al., 2024), enhancing regulatory efficiency and supporting equitable access, particularly for low- and middle-income countries (LMICs) that rely on reference-agency decisions for regulatory guidance (Akhade et al., 2022; Tibau et al., 2025).

This study has several limitations. First, using FDA-first approvals as the reference may bias the analysis toward U.S. practices. Although this approach is policy-relevant given the FDA’s leading role in initiating expedited oncology approvals, it does not capture alignment that may occur independently among the EMA, TGA, and PMDA. Second, as publicly available regulatory databases do not systematically disclose reasons for non-submission or rejection, the “not approved” category may encompass heterogeneous outcomes—including non-submission, ongoing review, and outright rejection—which could affect the interpretation of agency-level approval rates. However, this limitation is unlikely to materially affect the primary analytical concordance findings of this study. Our concordance framework (Levels 1–6) was applied only to drug–indication pairs that received subsequent approval by the comparator agencies, and thus excludes non-approved cases from the analysis of trial-level regulatory differences. Accordingly, the impact of this heterogeneity is primarily confined to the interpretation of approval patterns rather than the comparative assessment of regulatory decision-making among approved cases. Third, review duration includes both agency assessment time and any clock-stop periods during which the agency awaits sponsor responses, and therefore does not fully isolate agency efficiency from sponsor responsiveness. Moreover, because the study period overlapped with the COVID-19 pandemic, submission and approval timelines may also have been affected by external disruptions in clinical trial conduct, sponsor operations, and regulatory processes. Figure 2 may indicate a temporal gap in FDA approvals, which could be consistent with pandemic-related disruption or post-pandemic changes in global development and regulatory coordination. However, these patterns should be interpreted cautiously, as the present study was not designed to assess the causal impact of COVID-19, and robust comparison of the pre- and post-pandemic periods lies beyond the scope of the current analysis. This question therefore warrants more focused investigation in future research. Fourth, this study focused on initial regulatory decisions at the time of first marketing authorization and did not consider post-approval obligations, modifications or withdrawals. While these post-marketing changes are important in understanding the full regulatory lifecycle, our objective was to compare how agencies apply different regulatory standards to the same applications at the point of initial review. Nonetheless, this represents an important avenue for future research, where a more in-depth and systematic assessment of the post-approval regulatory landscape could be undertaken. Finally, this study focused on a single expedited approval designation per agency and excluded expedited review mechanisms. This restriction enhanced analytical clarity but may have omitted interactions between the approval and review processes—an area for future research.

Our findings highlight persistent divergence in regulatory frameworks and evidentiary thresholds across agencies, reflected in differences in data maturity requirements, approval pathways, and review timing that contribute to fragmented regulatory outcomes and unequal patient access. Greater transparency, aligned evidentiary standards, and strengthened international collaboration will be critical to realizing the full potential of expedited programs and promoting global regulatory harmonization.

## Data Availability

All relevant data presented in the study are included in the article/Supplementary Material, further inquiries can be directed to the corresponding authors.

## References

[B1] Agresti A. (1992). A survey of exact inference for contingency tables. Stat. Science 7, 131–153. 10.1214/ss/1177011454

[B2] Akhade A. Sirohi B. Gyawali B. (2022). Global consequences of the US FDA's accelerated approval of cancer drugs. Lancet Oncol. 23, 201–203. 10.1016/S1470-2045(21)00709-9 35114117

[B3] Beakes-Read G. Neisser M. Frey P. Guarducci M. (2022). Analysis of fDA's accelerated approval program performance December 1992-December 2021. Ther. Innov. Regul. Sci. 56, 698–703. 10.1007/s43441-022-00430-z 35900722 PMC9332089

[B4] Beaver J. A. Howie L. J. Pelosof L. Kim T. Liu J. Goldberg K. B. (2018). A 25-Year experience of US food and drug administration accelerated approval of malignant hematology and oncology drugs and biologics: a review. JAMA Oncol. 4, 849–856. 10.1001/jamaoncol.2017.5618 29494733

[B5] Benjamin D. J. Lythgoe M. P. (2023). Modernising the US FDA's accelerated approval pathway. Lancet Oncol. 24, 203–205. 10.1016/S1470-2045(23)00020-7 36858720

[B6] Centre for Innovation in Regulatory Science (Cirs) (2024). R&D briefing 93: new drug approvals in six major authorities 2014-2023: changing regulatory landscape and facilitated regulatory pathways. Centre Innovation Regul. Sci.

[B7] Croft M. D. A. (2018). “Implementation of data cut off in analysis of clinical trials,” in *PharmaSUG 2018.* PharmaSUG.

[B8] De Claro R. A. Spillman D. Hotaki L. T. Shum M. Mouawad L. S. Santos G. M. L. (2020). Project orbis: Global collaborative review program. Clin. Cancer Res. 26, 6412–6416. 10.1158/1078-0432.CCR-20-3292 33037016

[B9] Downing N. S. Aminawung J. A. Shah N. D. Krumholz H. M. Ross J. S. (2014). Clinical trial evidence supporting FDA approval of novel therapeutic agents, 2005-2012. Jama 311, 368–377. 10.1001/jama.2013.282034 24449315 PMC4144867

[B10] Drago D. Foss-Campbell B. Wonnacott K. Barrett D. Ndu A. (2021). Global regulatory progress in delivering on the promise of gene therapies for unmet medical needs. Mol. Ther. Methods Clin. Dev. 21, 524–529. 10.1016/j.omtm.2021.04.001 33997101 PMC8099595

[B11] European Medicines Agency (2019). EMA/FDA analysis shows high degree of alignment in marketing-application decisions between EU and US. Available online at: https://www.ema.europa.eu/en/news/ema-fda-analysis-shows-high-degree-of-alignment-marketing-application-decisions-between-eu-and-us (Accessed September 30, 2025).

[B12] European Medicines Agency (n.d.-a). Conditional marketing authorisation. Available online at: https://www.ema.europa.eu/en/human-regulatory-overview/marketing-authorisation/conditional-marketing-authorisation (Accessed June 9, 2025).

[B13] European Medicines Agency (n.d.-b). EMA medicines for human use Available online at: https://www.ema.europa.eu/en/medicines (Accessed September 30, 2025).

[B14] Forrest R. Lagarde M. Aggarwal A. Naci H. (2024). Preferences for speed of access versus certainty of the survival benefit of new cancer drugs: a discrete choice experiment. Lancet Oncol. 25, 1635–1643. 10.1016/S1470-2045(24)00596-5 39571597

[B15] Franco P. Jain R. Rosenkrands-Lange E. Hey C. Koban M. U. (2023). Regulatory pathways supporting expedited drug development and approval in ICH member countries. Ther. Innov. Regul. Sci. 57, 484–514. 10.1007/s43441-022-00480-3 36463352 PMC9734413

[B16] Freeman G. H. Halton J. H. (1951). Note on an exact treatment of contingency, goodness of fit and other problems of significance. Biometrika 38, 141–149. 10.1093/biomet/38.1-2.141 14848119

[B17] Holm S. (1979). A simple sequentially rejective multiple test procedure. Scand. J. Statistics 6, 65–70. 10.2307/4615733

[B18] Huang Y. Xiong W. Zhao J. Li W. Ma L. Wu H. (2023). Early phase clinical trial played a critical role in the food and drug Administration-approved indications for targeted anticancer drugs: a cross-sectional study from 2012 to 2021. J. Clin. Epidemiol. 157, 74–82. 10.1016/j.jclinepi.2023.03.006 36905971

[B19] Hwang T. J. Ross J. S. Vokinger K. N. Kesselheim A. S. (2020). Association between FDA and EMA expedited approval programs and therapeutic value of new medicines: retrospective cohort study. BMJ 371, m3434. 10.1136/bmj.m3434 33028575 PMC7537471

[B20] Hwang T. J. Kesselheim A. S. Tibau A. Lee C. C. Vokinger K. N. (2022). Clinical benefit and expedited approval of cancer drugs in the United States, european union, Switzerland, Japan, Canada, and Australia. JCO Oncol. Pract. 18, e1522–e1532. 10.1200/OP.21.00909 35731996 PMC9509186

[B21] Kashoki M. Hanaizi Z. Yordanova S. Vesely R. Bouygues C. Llinares J. (2020). A comparison of EMA and FDA decisions for new drug marketing applications 2014-2016: concordance, discordance, and why. Clin. Pharmacol. Ther. 107, 195–202. 10.1002/cpt.1565 31306483 PMC6977394

[B22] Knottnerus J. A. Knottnerus B. J. (2022). Decision-making given surrogate outcomes. J. Clin. Epidemiol. 145, 174–178. 10.1016/j.jclinepi.2022.01.003 35041971

[B23] Koole S. N. Huisman A. H. Timmers L. Westgeest H. M. Van Breugel E. Sonke G. S. (2024). Lessons learned from postmarketing withdrawals of expedited approvals for oncology drug indications. Lancet Oncol. 25, e126–e135. 10.1016/S1470-2045(23)00592-2 38423058

[B24] Kuhler T. C. Bujar M. Mcauslane N. Liberti L. (2019). To what degree are review outcomes aligned for new active substances (NASs) between the european medicines agency and the US food and drug administration? A comparison based on publicly available information for NASs initially approved in the time period 2014 to 2016. BMJ Open 9, e028677. 10.1136/bmjopen-2018-028677 31772082 PMC6887045

[B25] Lee S. Lee D. K. (2018). What is the proper way to apply the multiple comparison test? Korean J. Anesthesiol. 71, 353–360. 10.4097/kja.d.18.00242 30157585 PMC6193594

[B26] Liu I. T. T. Kesselheim A. S. Cliff E. R. S. (2024). Clinical benefit and regulatory outcomes of cancer drugs receiving accelerated approval. JAMA 331, 1471–1479. 10.1001/jama.2024.2396 38583175 PMC11000139

[B27] Lythgoe M. P. Desai A. Gyawali B. Savage P. Krell J. Warner J. L. (2022). Cancer therapy approval timings, review speed, and publication of pivotal registration trials in the US and Europe, 2010-2019. JAMA Netw. Open 5, e2216183. 10.1001/jamanetworkopen.2022.16183 35687337 PMC9187952

[B28] Manyara A. M. Davies P. Stewart D. Weir C. J. Young A. E. Wells V. (2023). Definitions, acceptability, limitations, and guidance in the use and reporting of surrogate end points in trials: a scoping review. J. Clin. Epidemiol. 160, 83–99. 10.1016/j.jclinepi.2023.06.013 37380118

[B29] Mehta G. U. De Claro R. A. Pazdur R. (2022). Accelerated approval is not conditional approval: insights from international expedited approval programs. JAMA Oncol. 8, 335–336. 10.1001/jamaoncol.2021.6854 35050302

[B30] Miyazaki T. Komiyama M. Matsumaru N. Maeda H. Tsukamoto K. (2022). Lag time for new innovative, First-in-Class, drug approval in Japan. Biol. Pharm. Bull. 45, 477–482. 10.1248/bpb.b21-00898 35370272

[B46] National Research Council (2010). The Prevention and Treatment of Missing Data in Clinical Trials. 24983040

[B31] National Cancer Institute (2025). Cancer Stat. Available online at: https://www.cancer.gov/about-cancer/understanding/statistics (Accessed June 4, 2025).

[B32] Patel G. Z. (2024). “Applying different data cutoff dates for the analysis of a study milestone,” in *SESUG 2024.* southeastern SAS users group (SESUG).

[B33] Perini M. Castiglioni B. Galai E. Trapani D. Genazzani A. A. Miglio G. (2025). Differences in the on-label cancer indications of medicinal products between Europe and the USA. Lancet Oncol. 26, e103–e110. 10.1016/S1470-2045(24)00434-0 39914420

[B34] Pharmaceutical Safety and Environmental Health Bureau Ministry of Health (2024). “Establishing a safe and expedited approval system to address challenges in access to pharmaceuticals, including drug lag and supply shortages,” in *Theme 1: establishing a safe and expedited approval system to address challenges in access to pharmaceuticals, including drug lag and supply shortages* .

[B35] Pharmaceuticals and Medical Devices Agency (n.d.). PMDA List of Approved Products [Online]. Available: https://www.pmda.go.jp/english/review-services/reviews/approved-information/drugs/0003.html (Accessed September 30, 2025).

[B36] Pharmaceuticals and Medical Devices Agency (2017). Regulatory update from MHLW/PMDA.

[B37] Ribeiro T. B. Colunga-Lozano L. E. Araujo A. P. V. Bennett C. L. Hozo I. Djulbegovic B. (2022). Single-arm clinical trials that supported FDA accelerated approvals have modest effect sizes and were at high risk of bias. J. Clin. Epidemiol. 148, 193–195. 10.1016/j.jclinepi.2022.01.018 35093531

[B38] Ribeiro T. B. Bennett C. L. Colunga-Lozano L. E. Araujo A. P. V. Hozo I. Djulbegovic B. (2023). Increasing FDA-Accelerated approval of single-arm trials in oncology (1992 to 2020). J. Clin. Epidemiol. 159, 151–158. 10.1016/j.jclinepi.2023.04.001 37037322

[B39] Rohr U. P. Iovino M. Rudofsky L. Li Q. Juritz S. Gircys A. (2023). A decade comparison of regulatory decision patterns for oncology products to all other non-oncology products among swissmedic, european medicines agency, and US food and drug administration. Clin. Transl. Sci. 16, 1569–1581. 10.1111/cts.13567 37408165 PMC10499418

[B40] Salcher-Konrad M. Naci H. Davis C. (2020). Approval of cancer drugs with uncertain therapeutic value: a comparison of regulatory decisions in Europe and the United States. Milbank Q. 98, 1219–1256. 10.1111/1468-0009.12476 33021339 PMC7772660

[B41] Schnog J. B. Samson M. J. Gans R. O. B. Duits A. J. (2021). An urgent call to raise the bar in oncology. Br. J. Cancer 125, 1477–1485. 10.1038/s41416-021-01495-7 34400802 PMC8365561

[B42] Sharma R. Gulati A. Chopra K. (2025). Era of surrogate endpoints and accelerated approvals: a comprehensive review on applicability, uncertainties, and challenges from regulatory, payer, and patient perspectives. Eur. J. Clin. Pharmacol. 81, 605–623. 10.1007/s00228-025-03822-w 40080138

[B43] Sumi E. Asada R. Lu Y. Ito-Ihara T. Grimes K. V. (2020). A qualitative study on the differences between trial populations and the approved therapeutic indications of antineoplastic agents by 3 regulatory agencies from 2010 to 2018. Clin. Ther. 42, 305–320 e300. 10.1016/j.clinthera.2020.01.002 32008723

[B44] Tafuri G. Stolk P. Trotta F. Putzeist M. Leufkens H. G. Laing R. O. (2014). How do the EMA and FDA decide which anticancer drugs make it to the market? A comparative qualitative study on decision makers' views. Ann. Oncol. 25, 265–269. 10.1093/annonc/mdt512 24356637

[B45] Tanaka M. Miyazawa H. Terashima R. Ikuma M. (2023). Conditional early approval for new drug applications in Japan: current and emerging issues. Clin. Transl. Sci. 16, 1289–1293. 10.1111/cts.13536 37161871 PMC10432865

[B47] Therapeutic Goods Administration (2018). Provisional approval pathway: prescription medicines. Available online at: https://www.tga.gov.au/provisional-approval-pathway-prescription-medicines (Accessed June 9, 2025).

[B48] Tibau A. Cliff E. R. S. Romano A. Borrell M. Molto C. Kesselheim A. S. (2025). Predictors of withdrawal of anticancer drug indications granted accelerated approval: a retrospective cohort study. EClinicalMedicine 84, 103088. 10.1016/j.eclinm.2025.103088 40687736 PMC12273735

[B49] Trotta F. Leufkens H. G. Schellens J. H. Laing R. Tafuri G. (2011). Evaluation of oncology drugs at the european medicines agency and US food and drug administration: when differences have an impact on clinical practice. J. Clin. Oncol. 29, 2266–2272. 10.1200/JCO.2010.34.1248 21537038

[B50] U.S. Food and Drug Administration (n.d.). Drugs@FDA Available online at: https://www.fda.gov/drugs (Accessed September 30, 2025).

[B51] U.S. Food and Drug Administration (2024). Accelerated approval program. Available online at: https://www.fda.gov/drugs/nda-and-bla-approvals/accelerated-approval-program (Accessed June 9, 2025).

[B52] U.S. Food and Drug Administration (2023). Novel drug approvals at. FDA. Available online at: https://www.fda.gov/drugs/development-approval-process-drugs/novel-drug-approvals-fda (Accessed June 30, 2024).

[B53] Vokinger K. N. Kesselheim A. S. Glaus C. E. G. Hwang T. J. (2022). Therapeutic value of drugs granted accelerated approval or conditional marketing authorization in the US and Europe from 2007 to 2021. JAMA Health Forum 3, e222685. 10.1001/jamahealthforum.2022.2685 36200635 PMC9391955

[B54] Xie J. Li J. Liu Y. Wang H. Wang Y. Yang Y. (2023). Comparison of novel oncology drugs that received dual approval from the US accelerated approval and EU conditional marketing authorisation pathways, 2006-2021: a cross-sectional study. BMJ Open 13, e069132. 10.1136/bmjopen-2022-069132 PMC10255285 37286329

[B55] Yoon J. Lee S. Kim M. J. Kim J. H. (2024). Brief summary of the regulatory frameworks of regenerative medicine therapies. Front. Pharmacol. 15, 1486812. 10.3389/fphar.2024.1486812 39911827 PMC11794276

[B56] Zeukeng M. J. Seoane-Vazquez E. Bonnabry P. (2018). A comparison of new drugs approved by the FDA, the EMA, and swissmedic: an assessment of the international harmonization of drugs. Eur. J. Clin. Pharmacol. 74, 811–818. 10.1007/s00228-018-2431-7 29470610

[B57] Zosso-Pavic M. Li Q. Atiek E. Wolfer A. Rohr U. P. (2024). Effect of project orbis participation by the Swiss regulator on submission gaps, review times, and drug approval decisions between 2020 and 2022: a comparative analysis. Lancet Oncol. 25, 770–778. 10.1016/S1470-2045(24)00158-X 38754450

