# Supplementary material for: FDA first to global follow-on: alignment in expedited oncology approvals across EMA, TGA, and PMDA
Source: Front Pharmacol. 2026 May 29;17:1804782. doi: 10.3389/fphar.2026.1804782 (PMC13260248; doi:10.3389/fphar.2026.1804782)
Supplement: Supplementary file 1 [file Table1.docx]

Supplementary Table 1. Comparison of expedited approval pathways across FDA, EMA, PMDA, and TGA

| **Jurisdiction**  **(Agency)** | **Expedited approval pathway** | **Year initiated** | **Purpose** | **Eligibility conditions** | **Basis for clinical benefit decision** | **Post-marketing requirements** |
| --- | --- | --- | --- | --- | --- | --- |
| United States  (FDA) | Accelerated Approval  (AA) | 1992 | Enable earlier approval of drugs for serious conditions based on surrogate or intermediate endpoints[1]. | Serious condition; meaningful advantage over existing therapies; effect on an endpoint reasonably likely to predict clinical benefit[1]. | Surrogate or intermediate endpoints reasonably likely to predict improvement in irreversible morbidity, mortality, or other clinical benefit[1]. | Mandatory confirmatory trials with FDA-specified timelines. Approval may be withdrawn if studies are not conducted with due diligence, fail to verify benefit, show unfavorable safety/effectiveness, or involve misleading promotion[1]. |
| European Union (EMA) | Conditional Market Authorisation  (CMA) | 2006 | Allow earlier access to treatments for seriously debilitating or life-threatening diseases when comprehensive data are pending[2]. | Positive benefit–risk; unmet medical need; likelihood of providing comprehensive post-authorization data; immediate availability outweighs uncertainties[2]. | Assessment of available evidence, including surrogate or intermediate endpoints reasonably likely to predict clinical benefit, with benefit–risk evaluated in context of incomplete data[2]. | Requires specific obligations and periodic safety updates. CMA validity is limited to 1 year and must be renewed annually based on fulfillment of obligations and updated benefit–risk. May be suspended/withdrawn if obligations are unmet or benefit–risk becomes unfavorable[2]. |
| Japan  (PMDA) | Conditional Early Approval  (CEA) | 2017 | Facilitate early access to high-utility drugs for severe diseases when confirmatory trials are impractical[3]. | Orphan, pioneering, or high-medical-need products for serious diseases; superiority to existing therapy or absence of alternatives; challenges in conducting confirmatory trials[4]. | Evidence from non-confirmatory clinical studies (e.g., exploratory studies, supportive non-clinical data) showing reasonable expectation of efficacy and safety[3]. | Requires use-results surveys, interim evaluations, and appropriate risk-management. CEA has no expiration date or fixed renewal requirement, and marketing continues as long as post-marketing obligations are fulfilled[4]. |
| Australia  (TGA) | Provisional approval | 2018 | Expedite access to promising medicines for serious or life-threatening conditions based on preliminary evidence[5]. | New medicine or new indication; favorable comparison to existing therapies; major therapeutic advance[6]. | Preliminary evidence may include non-validated surrogate endpoints, single-arm data, non-randomized comparisons, interim analyses, or limited sample sizes[6]. | Confirmatory trials must verify clinical benefit. Initial validity is 2 years, with extensions available up to a total of 6 years while required studies are completed. Failure to supply confirmatory data may prevent renewal or result in transition to full approval being denied[7, 8]. |

Abbreviations: FDA = US Food and Drug Administration; EMA = European Medicines Agency; TGA = Therapeutic Goods Administration; PMDA = Pharmaceuticals and Medical Devices Agency

Supplementary Methods. Definitions of Concordance

**Definition of Analytical Concordance Criteria**

To classify the degree of regulatory concordance across agencies following the FDA-first expedited oncology approvals, we assessed whether subsequent decisions were based on the same pivotal trial and whether key analytical components were interpreted consistently. Analytical concordance was evaluated across three components: (i) primary endpoint, (ii) target population, and (iii) data cut-off date (DCO). In the absence of clear divergence or where relevant information was not disclosed, cases were conservatively categorized as concordant.

**Evaluation Components and Criteria for Divergence**

(i) Primary Endpoint

- Criteria for Divergence: Divergence was identified when a regulatory agency explicitly designated a different primary efficacy endpoint from that used by the FDA.

(ii) Target Population

- Criteria for Divergence: Divergence was noted when an agency redefined or restricted the primary target population used in the FDA’s assessment. Specifically, the following modifications were considered indicative of analytical divergence:

-Restriction to a specific prior treatment history subgroup (e.g., line of therapy)

-Definition based on a biomarker or genetic profile

-Selection of specific treatment arms or dosing cohorts

-Limitation to a geographic or ethnic subgroup (e.g., inclusion of only Japanese patients within a global trial)

-Application of other population-defining criteria clearly stated in the agency’s review (e.g., censoring rules, cohort reassignment due to extended enrollment)

Note: The definition of the analysis population is directly linked to the determination of the approved indication. Therefore, criteria adapted from previous comparative regulatory studies were referred for consistency[9].

(iii) Data Cut-off Date (DCO)

- Criteria for Divergence: Divergence was defined by a discrepancy in the efficacy data cut-off dates (latest) used for the primary analysis, as identified in the agency’s public review documents compared to the FDA’s materials.

Supplementary Table 2. List of 36 oncology drug–indication pairs granted first expedited approval by FDA

| **No.** | **INN** | **Indication** | **WHO ATC code** | **Solid**  **/Hema** | **Line of therapy** | **Brand name** | **Company** | **Product type** | **Pharmacological class** | **Approval pathway (date)** | | | | **Comments** |
| --- | --- | --- | --- | --- | --- | --- | --- | --- | --- | --- | --- | --- | --- | --- |
|  |  |  |  |  |  |  |  |  |  | **FDA** | **EMA** | **TGA** | **PMDA** |  |
| 1 | Erdafitinib | Urothelial cancer | L01EN01 | Solid | 2 | Balversa | Janssen | NDA | small molecule targeted therapy | AA  (2019-04-12) | standard  (2024-08-22) | Not approved | Not approved |  |
| 2 | Polatuzumab vedotin | Diffuse Large B-cell Lymphoma | L01FX14 | Hema | 3 | Polivy | Genentech, Inc. | BLA | ADC | AA  (2019-06-10) | CMA  (2020-01-16) | Standard  (2019-10-21) | Standard  (2021-03-23) |  |
| 3 | Selinexor | Multiple myeloma | L01XX66 | Hema | 5 | Xpovio | KARYOPHARM | NDA | small molecule targeted therapy | AA  (2019-07-03) | CMA  (2021-03-26) | Standard  (2022-03-08) | Not approved |  |
| 4 | Zanubrutinib | Mantle cell lymphoma | L01EL03 | Hema | 2 | Brukinsa | BeiGene USA | NDA | small molecule targeted therapy | AA  (2019-11-14) | Not approved | Provisional  (2021-10-08) | Not approved | EMA marketing approval only for lymphoplasmacytic lymphoma, MZL, CLL, FL |
| 5 | Enfortumab vedotin | Urothelial cancer | L01FX13 | Solid | 3 | Padcev | Astellas Pharma US, Inc. | BLA | ADC | AA  (2019-12-18) | standard  (2022-04-13) | Standard  2022-07-07) | Standard  (2021-09-27) |  |
| 6 | Trastuzumab deruxtecan | Breast cancer | L01FD04 | Solid | 3 | Enhertu | Daiichi | BLA | ADC | AA  (2019-12-20) | CMA  (2021-01-18) | provisional  (2021-10-08) | CEA  (2020-03-25) |  |
| 7 | Tazemetostat | Epithelioid sarcoma | L01XX72 | Solid | 1 | Tazverik | Epizyme | NDA | small molecule targeted therapy | AA  (2020-01-23) | Not approved | Not approved | Not approved |  |
| 8 | Pemigatinib | Cholnagiocarcinoma | L01EN02 | Solid | 2 | Pemazyre | Incyte Corporation | NDA | small molecule targeted therapy | AA  (2020-04-17) | CMA  (2021-03-26) | Provisional  (2022-09-14) | Standard  (2021-03-23) |  |
| 9 | Sacituzumab govitecan | Breast cancer | L01FX17 | Solid | 3 | Trodelvy | Immunomedics | BLA | ADC | AA  (2020-04-22) | standard  (2021-11-22) | Standard  (2021-09-06) | Standard  (2024-09-24) |  |
| 10 | Capmatinib | Lung cancer | L01EX17 | Solid | 1 | Tabrecta | Novartis Pharmaceuticals Corporation | NDA | small molecule targeted therapy | AA  (2020-05-06) | standard  (2022-06-20) | Not approved | Standard  (2020-06-29) |  |
| 11 | Selpercatinib-1 | Lung cancer | L01EX22 | Solid | 1 | Retevmo | Loxo | NDA | small molecule targeted therapy | AA  (2020-05-08) | CMA  (2021-02-11) | Provisional  (2023-07-03 | Standard  (2021-09-27) |  |
| 12 | Selpercatinib-2 | Thyroid cancer | L01EX22 | Solid | 1 | Retevmo | Loxo | NDA | small molecule targeted therapy | AA  (2020-05-08) | CMA  (2021-02-11) | Not approved | Standard  (2022-02-25) |  |
| 13 | Selpercatinib-3 | Medullary Thyroid cancer | L01EX22 | Solid | 1 | Retevmo | Loxo | NDA | small molecule targeted therapy | AA  (2020-05-08) | CMA  (2021-02-11) | Not approved | Standard  (2022-02-25) |  |
| 14 | Lurbinectedin | Lung cancer | L01XX69 | Solid | 2 | Zepzelca | Pharma Mar USA | NDA | small molecule targeted therapy | AA  (2020-06-15) | Not approved | Provisional  (2021-09-13) | Not approved | EMA marketing authorization application submitted on 2025-05-21 |
| 15 | Tafasitamab | Diffuse Large B-cell Lymphoma | L01FX12 | Hema | 2 | Monjuvi | MorphoSys US | BLA | ADC | AA  (2020-07-31) | CMA  (2021-08-26) | provisional  (2023-06-20) | Not approved |  |
| 16 | Belantamab mafodotin | Multiple myeloma | L01XC39 | Hema | 5 | Blenrep | GlaxoSmithKline | BLA | ADC | AA  (2020-08-05) | CMA  (2020-08-25) | Not approved | Not approved |  |
| 17 | Pralsetinib | Lung cancer | L01EX23 | Solid | 1 | Gavreto | Blueprint Medicines Corporation | NDA | small molecule targeted therapy | AA  (2020-09-04) | CMA  (2021-11-18) | Provisional  (2023-03-29) | Not approved |  |
| 18 | Umbralisib-1 | Marignal zone lymphoma | L01EX25 | Hema | 2 | Ukoniq | TG Therapeutics | NDA | small molecule targeted therapy | AA  (2021-02-05) | Not approved | Not approved | Not approved |  |
| 19 | Umbralisib-2 | Follicular lymphoma | L01EX25 | Hema | 4 | Ukoniq | TG Therapeutics | NDA | small molecule targeted therapy | AA  (2021-02-05) | Not approved | Not approved | Not approved |  |
| 20 | Melphalan flufenamide | Multiple myeloma | L01AA10 | Hema | 5 | Pepaxto | Oncopeptides AB | NDA | Cytotoxic chemotherapy | AA  (2021-02-26) | standard  (2022-08-17) | Not approved | Not approved |  |
| 21 | Loncastuximab tesirine | Diffuse Large B-cell Lymphoma | L01FX22 | Hema | 3 | Zynlonta | ADC Therapeutics SA | BLA | ADC | AA  (2021-04-23) | CMA  (2022-12-20) | Not approved | Not approved |  |
| 22 | Amivantamab | Lung cancer | L01FX06 | Solid | 2 | Rybrevant | Janssen | BLA | Biologic therapy | AA  (2021-05-21) | CMA  (2021-12-09) | Provisional  (2022-12-01) | Not approved |  |
| 23 | Infigratinib | Cholnagiocarcinoma | L01EN03 | Solid | 2 | Truseltiq | QED Therapeutics | NDA | small molecule targeted therapy | AA  (2021-05-28) | Not approved | Provisional  (2021-11-05) | Not approved | EMA withdrawal of application |
| 24 | Sotorasib | Lung cancer | L01XX73 | Solid | 2 | Lumakras | Amgen | NDA | small molecule targeted therapy | AA  (2021-05-28) | CMA  (2022-01-06) | Provisional  (2022-03-30) | Standard  (2022-01-20) |  |
| 25 | Mobocertinib | Lung cancer | L01XX73 | Solid | 2 | Exkivity | Takeda | NDA | small molecule targeted therapy | AA  (2021-09-15) | Not approved | Provisional  (2022-07-19) | Not approved | EMA withdrawal of application |
| 26 | Tisotumab vedotin | Cervical cancer | L01FX23 | Solid | 2 | Tivdak | Seagen | BLA | ADC | AA  (2021-09-20) | Not approved | Not approved | Not approved | EMA marketing authorization issued on 2025-03-28 |
| 27 | Asciminib | Chronic myeloid leukemia (Ph+) | L01EA06 | Hema | 3 | Scemblix | Novartis | NDA | small molecule targeted therapy | AA  (2021-10-29) | standard  (2022-08-25) | Standard  (2022-07-15) | Standard  (2022-03-28) |  |
| 28 | Futibatinib | Cholangiocarcinoma | L01EN04 | Solid | 2 | Lytgobi | Taiho Oncology | NDA | Biologic therapy | AA  (2022-09-30) | CMA  (2023-07-04) | Not approved | Standard  (2023-06-26) |  |
| 29 | Mirvetuximab soravtansine | Epithelial ovarian, fallopian tube, or primary peritoneal cancer | L01FX26 | Solid | 4 | Elahere | ImmunoGen | BLA | ADC | AA  (2022-11-14) | standard  (2024-11-14) | Not approved | Not approved |  |
| 30 | Adagrasib | Lung cancer | L01XX77 | Solid | 2 | Krazati | Mirati Therapeutics | NDA | small molecule targeted therapy | AA  (2022-12-12) | CMA  (2024-01-05) | Not approved | Not approved |  |
| 31 | Pirtobrutinib | Mantle cell lymphoma | L01EL05 | Hema | 3 | Jaypirca | Loxo Oncology | NDA | small molecule targeted therapy | AA  (2023-01-27) | CMA  (2023-10-31) | Not approved | Standard  (2024-06-24) |  |
| 32 | Retifanlimab | Merkel cell carcinoma | L01FF10 | Solid | 1 | Zynyz | Incyte Corporation | BLA | biologic therapy | AA  (2023-03-22) | standard  (2024-04-19) | Not approved | Not approved |  |
| 33 | Epcoritamab | Diffuse Large B-cell Lymphoma | L01FX27 | Hema | 3 | Epkinly | Genmab US, Inc. | BLA | biologic therapy | AA  (2023-05-19) | CMA  (2023-09-22) | Provisional  (2025-01-06) | standard  2023-09-25) |  |
| 34 | Glofitamab | Diffuse Large B-cell Lymphoma | L01FX28 | Hema | 3 | Columvi | Genentech | BLA | biologic therapy | AA  (2023-06-15) | CMA  (2023-07-07) | provisional  (2023-08-09) | Not approved |  |
| 35 | Talquetamab | Multiple myeloma | L01FX29 | Hema | 5 | Talvey | Janssen | BLA | biologic therapy | AA  (2023-08-09) | CMA  (2023-08-21) | provisional  (2024-09-26) | Not approved |  |
| 36 | Elranatamab | Multiple myeloma | L01FX32 | Hema | 5 | Elrexfio | Pfizer | BLA | biologic therapy | AA  (2023-08-14) | CMA  (2023-12-07) | Provisional  (2024-06-28) | Standard  (2024-03-26) |  |

NOTE: This table summarizes 36 oncology drug–indication pairs that received first expedited approval by FDA between Jan 1, 2019, and Dec 31, 2023. It includes details on therapeutic class, product type, line of therapy, and timing of subsequent approvals by other agencies. Each subsequent regulatory decision is categorized by approval pathway (expedited vs standard) and date.

**Abbreviations:** AA = accelerated approval (FDA); ADC = antibody–drug conjugate; ATC = Anatomical Therapeutic Chemical classification; BLA = Biologics License Application; CEA = conditional early approval (PMDA); CMA = conditional marketing authorization (EMA); EMA = European Medicines Agency; FDA = US Food and Drug Administration; Hema = hematologic malignancy; NDA = New Drug Application; PMDA = Pharmaceuticals and Medical Devices Agency; TGA = Therapeutic Goods Administration

Supplementary Table 3. Pairwise fisher exact test *p*-values for comparisons of regulatory agency trial characteristics (FDA, EMA, TGA, PMDA)

| **Comparison** | **Characteristic** | **Unadjusted p-value** | **Holm-Bonferroni Adjusted p-value** |
| --- | --- | --- | --- |
| FDA vs. EMA | Randomization (RCT vs Non-RCT) | 0·22 | 1.00 |
| FDA vs. EMA | Phase (1 vs 1/2 vs 2 vs 3) | 0·33 | 1.00 |
| FDA vs. EMA | Target Population Size (<200 vs ≥200) | 0·018 | 0·11 |
| FDA vs. EMA | Primary Endpoint (OS vs Surrogate) | 0·17 | 1.00 |
| FDA vs. TGA | Randomization (RCT vs Non-RCT) | 0·46 | 1.00 |
| FDA vs. TGA | Phase (1 vs 1/2 vs 2 vs 3) | 0·44 | 1.00 |
| FDA vs. TGA | Target Population Size (<200 vs ≥200) | 0·036 | 0·18 |
| FDA vs. TGA | Primary Endpoint (OS vs Surrogate) | 0·33 | 1.00 |
| FDA vs. PMDA | Randomization (RCT vs Non-RCT) | 0·44 | 1.00 |
| FDA vs. PMDA | Phase (1 vs 1/2 vs 2 vs 3) | 0·42 | 1.00 |
| FDA vs. PMDA | Target Population Size (<200 vs ≥200) | 0·088 | 0·35 |
| FDA vs. PMDA | Primary Endpoint (OS vs Surrogate) | 0·32 | 1.00 |
| EMA vs. TGA | Randomization (RCT vs Non-RCT) | 1·00 | 1.00 |
| EMA vs. TGA | Phase (1 vs 1/2 vs 2 vs 3) | 1·00 | 1.00 |
| EMA vs. TGA | Target Population Size (<200 vs ≥200) | 1.00 | 1.00 |
| EMA vs. TGA | Primary Endpoint (OS vs Surrogate) | 1.00 | 1.00 |
| EMA vs. PMDA | Randomization (RCT vs Non-RCT) | 1.00 | 1.00 |
| EMA vs. PMDA | Phase (1 vs 1/2 vs 2 vs 3) | 0·54 | 1.00 |
| EMA vs. PMDA | Target Population Size (<200 vs ≥200) | 1.00 | 1.00 |
| EMA vs. PMDA | Primary Endpoint (OS vs Surrogate) | 1.00 | 1.00 |
| TGA vs. PMDA | Randomization (RCT vs Non-RCT) | 1.00 | 1.00 |
| TGA vs. PMDA | Phase (1 vs 1/2 vs 2 vs 3) | 0·51 | 1.00 |
| TGA vs. PMDA | Target Population Size (<200 vs ≥200) | 1.00 | 1.00 |
| TGA vs. PMDA | Primary Endpoint (OS vs Surrogate) | 1.00 | 1.00 |

NOTE: Pairwise comparisons between regulatory agencies were conducted using Fisher exact tests for each trial characteristic category. Both unadjusted and Holm-Bonferroni adjusted p-values are presented to account for multiple testing. These pairwise tests complement the overall Fisher–Freeman–Halton exact tests reported in Table 1 of the main manuscript. None of the pairwise comparisons reached statistical significance after adjustment, supporting findings from the overall tests.

Abbreviations: FDA = US Food and Drug Administration; EMA = European Medicines Agency; TGA = Therapeutic Goods Administration; PMDA = Pharmaceuticals and Medical Devices Agency; RCT = randomized controlled trial; OS = overall survival

**Supplementary Table 4. Pairwise fisher exact test *p*-values for distribution of concordance Levels 1–6 (EMA, TGA, PMDA)**

| **Comparison** | **Distribution** | **Unadjusted *p*-value** | **Holm-Bonferroni Adjusted p*-*value** |
| --- | --- | --- | --- |
| EMA vs. TGA | Concordance levels 1–6 | 0·13 | 0·13 |
| EMA vs. PMDA | Concordance levels 1–6 | 0·00014 | 0·00041 |
| TGA vs. PMDA | Concordance levels 1–6 | 0·0017 | 0·0033 |

Pairwise *p-*values were derived using Fisher’s exact test to compare differences in the distribution of regulatory concordance levels (Levels 1–6) between agencies. These pairwise tests complement the overall Fisher–Freeman–Halton exact tests reported in Table 2. Holm–Bonferroni correction was applied to adjust for multiple comparisons. Adjusted *P* < 0.05 was considered statistically significant; significant comparisons are shown in bold.

Abbreviations: EMA = European Medicines Agency; TGA = Therapeutic Goods Administration; PMDA = Pharmaceuticals and Medical Devices Agency

Supplementary Table 5. Pivotal trials and divergence patterns of Level 6 approvals

|  | **Drug**  **(Indication)** | **FDA Expedited Pivotal Trial** | **FDA Confirmatory Trial** | **Agency's Pivotal Trial(s)** | **Divergence Pattern** |
| --- | --- | --- | --- | --- | --- |
| EMA | Erdafitinib  (Urothelial) | BLC2001  (NCT02365597) | BLC3001 Cohort 1  (NCT03390504) | BLC3001 Cohort 1  (NCT03390504) | FDA confirmatory |
|  | Enfortumab  (Urothelial) | EV-201 cohort 1  (NCT03219333) | EV-301  (NCT03474107) | EV-301  (NCT03474107) | FDA confirmatory |
|  | Sacituzumab  (Breast) | IMMU-132-01  (NCT01631552) | IMMU-132-05  (NCT02574455) | IMMU-132-05  (NCT02574455) | FDA confirmatory |
|  | Mirvetuximab  (Ovarian) | SORAYA  (NCT04296890) | MIRASOL  (NCT04209855) | MIRASOL  (NCT04209855) | FDA confirmatory |
| TGA | Enfortumab  (Urothelial) | EV-201 cohort 1  (NCT03219333) | EV-301  (NCT03474107) | EV-301  (NCT03474107) | FDA confirmatory |
|  | Sacituzumab  (Breast) | IMMU-132-01  (NCT01631552) | IMMU-132-05  (NCT02574455) | IMMU-132-05  (NCT02574455) | FDA confirmatory |
| PMDA | Enfortumab  (Urothelial) | EV-201 cohort 1  (NCT03219333) | EV-301  (NCT03474107) | EV-301  (NCT03474107) | FDA confirmatory |
|  | Sacituzumab  (Breast) | IMMU-132-01  (NCT01631552) | IMMU-132-05  (NCT02574455) | IMMU-132-05  (NCT02574455) | FDA confirmatory + Japanese |
|  | Epcoritamab  (DLBCL) | EPCORE™ NHL-1  (NCT03625037) | NA | EPCORE™ NHL-1 (NCT03625037) + EPCORE™ NHL-3* (NCT04542824) | Original + Japanese |
|  | Polatuzumab  (DLBCL) | GO29365  (NCT02257567) | NA | GO29365 (NCT02257567) + JO40762/P-DRIVE (JapicCTI-184048)* | Original + Japanese |

NOTE: This table presents drug–indication pairs that were categorized as Level 6, reflecting substantial divergence from FDA-first expedited approval. For each case, the pivotal trial supporting FDA’s initial approval and confirmatory trial (if applicable) are listed alongside the trial(s) cited by the EMA, TGA, or PMDA. All Level 4 cases involved the use of a different pivotal trial than the one employed by the FDA.

* PMDA-required pivotal trial conducted in Japan.

Abbreviations: DLBCL = diffuse large B-cell lymphoma; FDA = US Food and Drug Administration; EMA = European Medicines Agency; TGA = Therapeutic Goods Administration; PMDA = Pharmaceuticals and Medical Devices Agency NA = not applicable.

Supplementary Table 6. Cross-agency Comparison of Analytical Components for Matched Pivotal Trials (Level 1-2, 4-5)

| **INN** | **indication** | **Pivotal trial ID** | **Agency** | **3 Analysis Components** | | | | | |
| --- | --- | --- | --- | --- | --- | --- | --- | --- | --- |
|  |  |  |  | **(i) Primary endpoint** | **Divergence** | **(ii) Efficacy analysis population summary** | **Divergence** | **(iii) DCO date** | **Divergence** |
| Polatuzumab vedotin | Diffuse Large B-cell Lymphoma | NCT02257567 | FDA | CR | Reference | 80 patients with R/R DLBCL randomized to P+BR (n=40) or BR (n=40) | Reference | 2018-04-30 | Reference |
|  |  |  | EMA | CR | NA | 80 patients with R/R DLBCL randomized to P+BR (n=40) or BR (n=40) | NA | 2018-04-30 | NA |
|  |  |  | TGA | CR | NA | 80 patients with R/R DLBCL randomized to P+BR (n=40) or BR (n=40) | NA | 2018-04-30 | NA |
| Selinexor | Multiple myeloma | NCT02336815 | FDA | ORR | Ref | 122 patients with triple-class refractory MM | Ref | 2018-04-24 | Reference |
|  |  |  | EMA | ORR | NA | 83 penta-refractory patients | Prior treatment | 2019-09-07 | Extended |
|  |  |  | TGA | ORR | NA | 122 patients with triple-class refractory MM | NA | 2019-09-07 | Extended |
| Zanubrutinib | Mantle cell lymphoma | NCT03206970-china | FDA | ORR | Reference | 86 patients with centrally confirmed mantle cell lymphoma and measurable disease at baseline; received >=1 dose of zanubrutinib at 160 mg twice daily | Reference | 2019-02-15 | Reference |
|  |  |  | TGA | ORR | NA | 86 patients with centrally confirmed mantle cell lymphoma (MCL) and measurable disease at baseline; received >=1 dose of zanubrutinib at 160 mg twice daily | NA | 2020-09-08 | Extended |
|  |  | NCT02343120 | FDA | ORR | Reference | 32 previously treated MCL patients who received a total daily zanubrutinib dose of 320 mg (either 160 mg BID or 320 mg QD) | Reference | 2018-12-13 | Reference |
|  |  |  | TGA | ORR | NA | 37 previously treated MCL patients treated at the recommended Phase 1/2 zanubrutinib dose of 160 mg BID | Treatment regimen (TGA’s selection of the recommended Phase 1/2 dose cohort - the regimen most likely to be used in Australian clinical practice) | 2018-12-13 | NA |
| Trastuzumab deruxtecan | Breast cancer | NCT03248492 | FDA | ORR | Reference | 184 HER2+ female patients previously treated with ≥2 HER2-based regimens | Reference | 2019-03-21 | Reference |
|  |  |  | EMA | ORR | NA | 184 HER2+ female patients previously treated with ≥2 HER2-based regimens | NA | 2020-06-08 | Extended |
|  |  |  | PMDA | ORR | NA | 167 patients (≥1 dose + ICR-evaluable lesions); Japanese subgroup (n=30) analyzed | Geographic subgroup (Japan); Evaluability refinement | 2019-03-21 | NA |
|  |  |  | TGA | ORR | NA | 184 HER2+ female patients previously treated with ≥2 HER2-based regimens | NA | 2021-03-21 | Extended |
| Pemigatinib | Cholnagiocarcinoma | NCT02924376 | FDA | ORR | Reference | 107 patients with FGFR2 fusion/rearrangement-positive cancer treated with at least one dose of Pemigatinib (Cohort A) | Reference | 2019-03-22 | Reference |
|  |  |  | EMA | ORR | NA | 107 patients with FGFR2 fusion/rearrangement-positive cancer treated with at least one dose of Pemigatinib (Cohort A) | NA | 2020-04-07 | Extended |
|  |  |  | PMDA | ORR | NA | 107 patients with FGFR2 fusion/rearrangement-positive cancer treated with at least one dose of Pemigatinib (Cohort A) | NA | 2019-03-22 | NA |
|  |  |  | TGA | ORR | NA | 107 patients with FGFR2 fusion/rearrangement-positive cancer treated with at least one dose of Pemigatinib (Cohort A) | NA | 2020-04-07 | Extended |
| Capmatinib | Lung cancer | NCT02414139 | FDA | ORR | Reference | 97 patients with MET exon14-mutated cancer from Cohort 4 (pre-treated, n=69) and Cohort 5b (treatment-naïve, n=28) | Reference | 2019-10-28 | Reference |
|  |  |  | EMA | ORR | NA | 100 pre-treated patients with MET exon14-mutated cancer from Cohort 4 (n=69) and Cohort 6 (n=31) | Treatment regimen (Different cohort, line of therapy) | 2021-08-30 | Extended |
|  |  |  | PMDA | ORR | NA | 97 patients with MET exon14-mutated cancer from Cohort 4 (pre-treated, n=69) and Cohort 5b (treatment-naïve, n=28) + 13 Japanese patients with MET exon14-mutated cancer from Cohort 4 and Cohort 5b (n=11 and n=2, respectively; regional cohort divergence) | Geographic subgroup (Japan); Evaluability refinement | 2019-04-15 | NA |
| Selpercatinib-1 | Lung cancer | NCT03157128 | FDA | ORR | Reference | 39 treatment-naïve patients with RET fusion-positive NSCLC treated with Selpercatinib at 200 mg daily dose + Subset of the broader PAS 105 patient population, specifically focusing on patients who had not received prior systemic therapy | Reference | 2019-12-16 | Reference |
|  |  |  | EMA | ORR | NA | 105 consecutively enrolled patients with RET fusion-positive NSCLC previously treated with platinum-based chemotherapy (PAS) + Integrated Analysis Set (IAS) including all 218 patients with prior platinum-chemotherapy treatment | Broader target population | 2020-03-30 | Extended |
|  |  |  | PMDA | ORR | NA | 134 patients (25 Japanese patients analysis included) from Cohort 1 Prior platinum-based chemotherapy ± immunotherapy; treated at the recommended phase 2 dose + 35 patients (1 Japanese patient analysis included) from Cohort 2: No prior systemic therapy | Broader target population + Geographic subgroup (Japan) | 2020-03-30 | Extended |
|  |  |  | TGA | ORR | NA | 249 patients Prior platinum-based chemotherapy/immunotherapy; treated at RP2D + 69 patients treatment-naïve (No prior systemic therapy) | Broader target population | 2021-06-15 | Extended |
| Selpercatinib-2 | Thyroid cancer | NCT03157128 | FDA | ORR | Reference | 27 patients with RET fusion-positive thyroid cancer, including 19 systemic-therapy-naïve (first-line) and 8 previously treated (second-line) patients | Reference | 2019-12-16 | Reference |
|  |  |  | EMA | ORR | NA | 19 patients with RET fusion-positive thyroid cancer previously treated with systemic therapy (second-line population) | Treatment regimen (line of therapy) | 2020-03-30 | Extended |
|  |  |  | PMDA | ORR | NA | 22 patients, subdivided post hoc into 10 systemic-therapy–naïve and 12 previously treated, but without separate regulatory cohorts. | Treatment regimen (Different cohort) | 2020-03-30 | Extended |
| Selpercatinib-3 | Medullary Thyroid cancer | NCT03157128 | FDA | ORR | Reference | 88 patients with medullary thyroid cancer (MTC) and no prior RET-targeted MKI therapy; treated with Selpercatinib at 160 mg twice daily (SAS1 population) + 55 consecutively enrolled patients with MTC previously treated with Vandetanib and/or Cabozantinib; treated at the recommended Phase 2 dose (RP2D) of 160 mg twice daily (PAS population) | Reference | 2019-12-16 | Reference |
|  |  |  | EMA | ORR | NA | 55 consecutively enrolled patients with medullary thyroid cancer previously treated with vandetanib and/or Cabozantinib; treated at the recommended Phase 2 dose (RP2D) of 160 mg twice daily (PAS population) + 143 patients with MTC who had ≥6 months of follow-up at the data cut-off, including both RET-MKI-naïve and RET-MKI-pretreated patients (IAS population) | Broader target population | 2020-03-30 | Extended |
|  |  |  | PMDA | ORR | NA | 97 patients with RET-mutant medullary thyroid cancer from Cohort 3 who had received prior treatment with Cabozantinib or Vandetanib + 90 patients with RET-mutant MTC from Cohort 4 with no prior treatment with RET-targeted tyrosine kinase inhibitors (TKIs) | Treatment regimen (Different cohort) | 2020-03-30 | Extended |
| Lurbinectedin | Lung cancer | NCT02454972 | FDA | ORR | Reference | 105 patients with pretreated advanced solid tumors (SCLC, H&N carcinoma, etc.) | Reference | 2019-01-15 | Reference |
|  |  |  | TGA | ORR | NA | 105 patients with pretreated advanced solid tumors (SCLC, H&N carcinoma, etc.) | NA | 2019-01-15 | NA |
| Tafasitamab | Diffuse Large B-cell Lymphoma | NCT02399085 | FDA | ORR | Reference | 71 patients with central pathology-confirmed DLBCL (restricted from full set) | Reference | 2018-11-30 | Reference |
|  |  | NCT02399085 | EMA | ORR | NA | 81 patients with R/R DLBCL treated with Tafasitamab plus lenalidomide | Broader target population | 2020-10-30 | Extended |
|  |  | NCT02399085 | TGA | ORR | NA | 81 patients with R/R DLBCL treated with Tafasitamab plus lenalidomide | Broader target population | 2018-11-30 | NA |
| Belantamab mafodotin | Multiple myeloma | NCT03525678 | FDA | ORR | Reference | 97 and 99 patients with relapsed/refractory multiple myeloma who had received ≥4 prior therapies and were refractory to a proteasome inhibitor, an immunomodulatory agent, and an anti-CD38 mAb; treated with 2.5 or 3.4 mg/kg IV every 3 weeks (Day 1, 21-day cycle). | Reference | 2019-06-21 | Reference |
|  |  |  | EMA | ORR | NA | 97 and 99 patients with relapsed/refractory multiple myeloma who had received ≥4 prior therapies and were refractory to a proteasome inhibitor, an immunomodulatory agent, and an anti-CD38 mAb; treated with 2.5 or 3.4 mg/kg IV every 3 weeks (Day 1, 21-day cycle). | NA | 2020-01-31 | Extended |
| Pralsetinib | Lung cancer | NCT03037385 | FDA | ORR | Reference | 114 patients with RET fusion-positive metastatic NSCLC, including 87 previously treated with platinum-based chemotherapy and 27 treatment-naïve patients | Reference | 2020-05-22 | Reference |
|  |  |  | EMA | ORR | NA | 233 patients with advanced cancer, including 75 treatment-naïve, 136 previously treated with platinum-based therapy, and 22 previously treated with non-platinum systemic therapy | Broader target population | 2020-11-06 | Extended |
|  |  |  | TGA | ORR | NA | 114 patients with RET fusion-positive metastatic NSCLC, including 87 previously treated with platinum-based chemotherapy and 27 treatment-naïve patients | NA | 2020-05-22 | Extended |
| Melphalan flufenamide | Multiple myeloma | NCT02963493 | FDA | ORR | Reference | 97 patients who had received ≥4 prior lines of therapy and were refractory to ≥1 PI, ≥1 IMiD, and ≥1 CD38-targeted monoclonal antibody | Reference | 2019-07-30 | Reference |
|  |  |  | EMA | ORR | NA | 157 patients with R/R multiple myeloma, including 119 refractory or intolerant to ≥1 proteasome inhibitor (PI), ≥1 immunomodulatory drug (IMiD), and ≥1 CD38-targeted monoclonal antibody | Broader target population | 2021-08-12 | Extended |
| Loncastuximab tesirine | Diffuse Large B-cell Lymphoma | NCT03589469 | FDA | ORR | Reference | 145 patients with relapsed or refractory large B‐ell lymphoma who received at least one dose of Loncastuximab tesirine | Reference | 2020-04-06 | Reference |
|  |  |  | EMA | ORR | NA | 145 patients with relapsed or refractory large B‐cell lymphoma who received at least one dose of Loncastuximab tesirine | NA | 2021-03-01 | Extended |
| Amivantamab | Lung cancer | NCT02609776 | FDA | ORR | Reference | 81 patients with EGFR exon 20 ins NSCLC previously treated with chemotherapy | Reference | 2020-10-08 | Reference |
|  |  |  | EMA | ORR | NA | 114 patients (Exon 20ins, platinum-pretreated, RP2D dose, recent metastases) | Broader target population | 2021-03-30 | Extended |
|  |  |  | TGA | ORR | NA | 81 patients with EGFR exon 20 ins NSCLC previously treated with chemotherapy | Reference | 2021-03-30 | Extended |
| Infigratinib | Cholnagiocarcinoma | NCT02150967 | FDA | ORR | Reference | 108 patients with previously treated, unresectable locally advanced or metastatic cholangiocarcinoma with an FGFR2 fusion or other rearrangement | Reference | 2020-03-31 | Reference |
|  |  |  | TGA | ORR | NA | 108 patients with previously treated, unresectable locally advanced or metastatic cholangiocarcinoma with an FGFR2 fusion or other rearrangement | NA | 2020-03-31 | NA |
| Sotorasib | Lung cancer | NCT03600883 | FDA | ORR | Reference | 124 subjects who received ≥1 dose of Sotorasib and have ≥1 measurable lesion at baseline as assessed by BICR using RECIST 1.1; subjects with KRAS p.G12C-mutated advanced or metastatic NSCLC | Reference | 2020-12-01 | Reference |
|  |  |  | EMA | ORR | NA | 124 subjects who received ≥1 dose of Sotorasib and have ≥1 measurable lesion at baseline as assessed by BICR using RECIST 1.1; subjects with KRAS p.G12C-mutated advanced or metastatic NSCLC | NA | 2021-06-21 | Extended |
|  |  |  | PMDA | ORR | NA | 123 patients with KRAS G12C-mutated NSCLC from Part II of the study, including 10 Japanese patients; included in the full analysis set (FAS) after central confirmation of mutation and baseline measurable lesions by BICR | Geographic subgroup (Japan) | 2020-09-01 | Shorter |
|  |  |  | TGA | ORR | NA | 124 subjects who received ≥1 dose of Sotorasib and have ≥1 measurable lesion at baseline as assessed by BICR using RECIST 1.1; subjects with KRAS p.G12C-mutated advanced or metastatic NSCLC | NA | 2020-12-01 | NA |
| Mobocertinib | Lung cancer | NCT02716116 | FDA | ORR | Reference | 114 patients with NSCLC harboring EGFR exon 20 insertion mutations previously treated with platinum-based chemotherapy | Reference | 2020-11-01 | Reference |
|  |  |  | TGA | ORR | NA | 114 patients with NSCLC harboring EGFR exon 20 insertion mutations previously treated with platinum-based chemotherapy | NA | 2020-11-01 | NA |
| Asciminib | Chronic myeloid leukemia (Ph+) | NCT03106779 | FDA | MMR at 24 weeks | Reference | 233 patients with chronic myeloid leukemia randomized to Asciminib monotherapy (n=157) or bosutinib monotherapy (n=76) | Reference | 2020-05-25 | Reference |
|  |  |  | EMA | MMR at 24 weeks | NA | 233 patients with chronic myeloid leukemia randomized to Asciminib monotherapy (n=157) or bosutinib monotherapy (n=76) | NA | 2021-10-06 | Extended |
|  |  |  | PMDA | MMR at 24 weeks | NA | 233 patients with chronic myeloid leukemia randomized to Asciminib monotherapy (n=157) or bosutinib monotherapy (n=76) | NA | 2021-01-06 | Extended |
|  |  |  | TGA | MMR at 24 weeks | NA | 233 patients with chronic myeloid leukemia randomized to Asciminib monotherapy (n=157) or bosutinib monotherapy (n=76) | NA | 2021-10-06 | Extended |
| Futibatinib | Cholnagiocarcinoma | NCT02052778 | FDA | ORR | Reference | 103 patients with iCCA with FGFR2 rearrangements (including gene fusions) after progression on at least 1 prior line of systemic therapy with gemcitabine plus cisplatin. | Reference | 2020-10-01 | Reference |
|  |  |  | EMA | ORR | NA | 103 patients with iCCA with FGFR2 rearrangements (including gene fusions) after progression on at least 1 prior line of systemic therapy with gemcitabine plus cisplatin. | NA | 2022-05-29 | Extended |
|  |  |  | PMDA | ORR | NA | 103 patients with FGFR2-rearranged intrahepatic cholangiocarcinoma (iCCA), including gene fusions, after progression on ≥1 prior line of systemic therapy with gemcitabine plus cisplatin; includes 14 Japanese patients | Geographic subgroup (Japan) | 2022-05-29 | Extended |
| Adagrasib | Lung cancer | NCT03785249 | FDA | ORR | Reference | 112 patients in Cohort A, measurable disease at baseline by BICR, received >=1 dose | Reference | 2021-10-15 | Reference |
|  |  |  | EMA | ORR | NA | 116 enrolled patients in Cohort A, baseline eligibility by BICR | Broader target population; Evaluability refinement | 2022-01-15 | Extended |
| Pirtobrutinib | Mantle cell lymphoma | NCT03740529 | FDA | ORR | Reference | 120 patients from the PAS + SAS1 populations who were treated with the 200mg starting dose and either did not undergo dose escalation or underwent dose escalation only after an IRC-assessed PD event or permanent IRC censoring. | Reference | 2022-01-31 | Reference |
|  |  |  | EMA | ORR | NA | 90 subjects (PAS) with MCL enrolled from either phase 1 or phase 2, irrespective of Pirtobrutinib starting dose, who had received a prior BTK inhibitor-containing regimen | Original primary efficacy cohort as designed | 2022-07-29 | Extended |
|  |  |  | PMDA | ORR | NA | 65 patients (in the order of the start of treatment) with non-blastoid MCL treated with a prior BTK inhibitor-containing regimen (33 subjects from the phase I part and 32 subjects from the phase II part) (J-PAS, Japan-Primary Analysis Set) | Narrower target population redefined for the Japanese regulatory review to minimize potential bias | Not disclosed | NA |
| Retifanlimab | Merkel cell carcinoma | NCT03599713 | FDA | ORR | Reference | 65 chemotherapy-naïve participants who received at least 1 dose of Retifanlimab | Reference | 2022-01-21 | Reference |
|  |  |  | EMA | ORR | NA | 101 chemotherapy-naïve participants who received at least 1 dose of Retifanlimab | Due to extended enrollment | 2023-03-10 | Extended |
| Epcoritamab | Diffuse Large B-cell Lymphoma | NCT03625037 | FDA | ORR | Reference | 157 patients with relapsed/refractory LBCL after ≥2 prior systemic therapies; treated with Epcoritamab monotherapy (aNHL cohort) | Reference | 2022-06-30 | Reference |
|  |  |  | EMA | ORR | NA | 157 patients with relapsed/refractory LBCL after ≥2 prior systemic therapies; treated with Epcoritamab monotherapy (aNHL cohort), including 139 patients with DLBCL | Broader target population | 2022-06-30 | NA |
| Glofitamab | Diffuse Large B-cell Lymphoma | NCT03075696 | FDA | ORR | Reference | 132 patients with relapsed/refractory DLBCL not otherwise specified and trFL from pooled cohorts D2, subcohort 2, D3, and D5; after >=2 prior systemic therapies; received>=1 dose at the recommended schedule | Reference | 2022-06-15 | Reference |
|  |  |  | EMA | CR | Stricter measure of efficacy | 108 patients with relapsed/refractory DLBCL after >=2 prior therapies from Cohort D3; received Glofitamab at 2.5/10/30 mg every 3 weeks (Q3W); registrational dose population | Treatment regimen (single cohort) | 2022-06-15 | NA |
|  |  |  | TGA | CR | Stricter measure of efficacy | 132 patients with relapsed/refractory DLBCL not otherwise specified (NOS) and transformed follicular lymphoma (trFL) from pooled cohorts D2, subcohort 2, D3, and D5; after >=2 prior systemic therapies; received >=1 dose at the recommended schedule | NA | 2022-06-15 | NA |
| Talquetamab | Multiple myeloma | NCT04634552 | FDA | ORR | Reference | 187 patients from Phase 2 Cohorts A (0.4 mg/kg QW; n = 100) and C (0.8 mg/kg Q2W; n = 87) treated at the recommended Phase 2 doses | Reference | 2022-09-12 | Reference |
|  |  |  | EMA | ORR | NA | All 288 patients in Cohorts A and C (0.4 mg/kg QW and 0.8 mg/kg Q2W), pooled across Phase 1 and Phase 2 | Broader target population | 2023-01-17 | Extended |
| Elranatamab | Multiple myeloma | NCT04649359 | FDA | ORR | Reference | 97 BCMA-naïve Cohort A patients; received ≥4 prior lines including a proteasome inhibitor, an immunomodulatory agent, and an anti-CD38 antibody | Reference | 2022-10-14 | Reference |
|  |  |  | EMA | ORR | NA | all 123 BCMA-naïve Cohort A patients BCMA-naïve; no requirement on number of prior lines beyond being triple-class exposed (PI, IMiD, anti-CD38) | Broader target population | 2023-04-16 | Extended |
|  |  |  | PMDA | ORR | NA | all 123 BCMA-naïve Cohort A patients BCMA-naïve; no requirement on number of prior lines beyond being triple-class exposed (PI, IMiD, anti-CD38) | Broader target population | 2022-10-14 | NA |
|  |  |  | TGA | ORR | NA | all 123 BCMA-naïve Cohort A patients BCMA-naïve; no requirement on number of prior lines beyond being triple-class exposed (PI, IMiD, anti-CD38) | Broader target population | 2023-04-16 | Extended |

Note: This table summarizes differences in regulatory interpretation across FDA, EMA, TGA, and PMDA for drug–indication pairs sharing the same pivotal trial ID. Concordance or divergence is reported for the primary efficacy endpoint, the analysis population definition, and the data cut-off (DCO) date used in the primary assessment. Divergence is categorized when a regulatory agency deviated from the FDA in its analytical approach or evidentiary emphasis;

Divergence descriptions reflect differences in:

(i) Primary endpoint

- NA: No difference or data not disclosed.

(ii) Target population:

- Broader target population refers to inclusion of additional cohorts or patients beyond those analyzed by the FDA, often to increase statistical power or reflect real-world conditions.

- Geographic subgroup divergence denotes when a region-specific subset (e.g., Japanese patients) was analyzed separately by a non-FDA agency, even if the trial was global.

- Evaluability refinement includes changes such as reassignment of patient cohorts or inclusion/exclusion based on measurable lesion status, central pathology confirmation, or imaging review.

- Treatment regimen divergence refers to reliance on different dosing cohorts, lines of therapy, or cohort phases between agencies.

- NA: No difference or data not disclosed.

(iii) Data cut-off (DCO) date: e.g., extended follow-up or updated datasets at the time of submission.

- Extended: The agency used a later data cut-off than the FDA.

- Shorter: The agency used an earlier cut-off than the FDA.

- NA: No difference or data not disclosed.

Abbreviations: BICR = blinded independent central review; BR = bendamustine plus rituximab; CML = chronic myeloid leukemia; CR = complete response; DCO = data cut-off; DLBCL = diffuse large B-cell lymphoma; RR DLBCL=Relapsed-Refractory Diffuse Large B Cell Lymphoma; FGFR2 = fibroblast growth factor receptor 2; HER2 = human epidermal growth factor receptor 2; H&N=head and neck; iCCA = intrahepatic cholangiocarcinoma; IMiD = immunomodulatory drug; IRC = independent review committee; IV = intravenous; MCL = mantle cell lymphoma; MKI = multi-kinase inhibitor; MM = multiple myeloma; MMR = major molecular response; MTC = medullary thyroid cancer; NSCLC = non-small cell lung cancer; ORR = objective response rate; PAS = primary analysis set; PI = proteasome inhibitor; PMDA = Pharmaceuticals and Medical Devices Agency; QW = once weekly; Q2W = once every 2 weeks; QD = once daily; BID = twice daily; RECIST = Response Evaluation Criteria in Solid Tumors; RP2D = recommended phase 2 dose; R/R = relapsed or refractory; SAS = supplemental analysis set; SCLC = small cell lung cancer; TGA = Therapeutic Goods Administration; trFL = transformed follicular lymphoma

Supplementary Table 7. Data cut-off date intervals by agency and concordance level (in days)

|  | **EMA** | | | **TGA** | | | **PMDA** | | |
| --- | --- | --- | --- | --- | --- | --- | --- | --- | --- |
| **Level** | **1-2** | **4-5** | **Total** | **1-2** | **4-5** | **Total** | **1-2** | **4-5** | **Total** |
| n | 20 | 4 | 24 | 13 | 3 | 16 | 1 | 10 | 11 |
| Min | 0 | 413 | 0 | 0 | 0 | 0 | 0 | -196 | -196 |
| Q1 | 105 | 478 | 105 | 0 | 250 | 0 | 0 | 0 | 0 |
| Median | 176 | 586 | 193 | 0 | 499 | 87 | 0 | 52.5 | 0 |
| Q3 | 356 | 690 | 458 | 382 | 500 | 500 | 0 | 105 | 105 |
| Max | 700 | 744 | 744 | 731 | 501 | 731 | 0 | 605 | 605 |
| p-value |  | 0·015 |  |  | 0·62 |  |  | 0·74 |  |

NOTE: This table summarizes data cut-off date intervals, in days, for regulatory approvals based on the same pivotal trial as that used by the FDA, stratified by concordance level within the six-level framework. Levels 1–2 denote subsequent expedited approvals on the same pivotal trial basis as the FDA, whereas Levels 4–5 denote subsequent standard approvals on the same pivotal trial basis; the “Total” column aggregates Levels 1–2 and Levels 4–5.

Negative values indicate that the subsequent agency’s analysis relied on an earlier DCO date than that used by the FDA. Data are presented for the EMA, TGA, and PMDA, with descriptive statistics including quartiles (Q1, median, Q3) and minimum, maximum for each group.

p-values were derived from Wilcoxon rank-sum tests comparing Levels 1–2 versus 4–5 within each regulatory agency. p < 0·05 was considered statistically significant.

Abbreviations: FDA = US Food and Drug Administration; EMA = European Medicines Agency; TGA = Therapeutic Goods Administration; PMDA = Pharmaceuticals and Medical Devices Agency

**Supplementary Reference**

1. U.S. Food and Drug Administration; CDER; CBER; Oncology Center of Excellence. Expedited Program for Serious Conditions — Accelerated Approval of Drugs and Biologics: Guidance for Industry (Draft). 2024 December 2024.

2. European Medicines Agency; Committee for Medicinal Products for Human Use. Guideline on the scientific application and the practical arrangements necessary to implement Commission Regulation (EC) No 507/2006 on the conditional marketing authorisation for medicinal products for human use falling within the scope of Regulation (EC) No 726/2004 (Draft). European Medicines Agency; 2015 23 July 2015.

3. Shibatsuji M. Introduction of the Conditional Early Approval (CEA) System in Japan. The 7th Asia Partnership Conference of Pharmaceutical Associations (APAC); 2018-04-102018.

4. Pharmaceutical Safety and Environmental Health Bureau Ministry of Health L.a.W. Establishing a Safe and Expedited Approval System to Address Challenges in Access to Pharmaceuticals, including Drug Lag and Supply Shortages. 2024 2024-6-6.

5. Administration T.G. Apply for prescription medicine provisional registration pathway: Australian Government Department of Health and Aged Care; n.d. [updated 1 May 2025. Available from: <https://www.tga.gov.au/products/medicines/prescription-medicines/application-and-market-authorisation/supply-prescription-medicine/application-process-prescription-medicines/apply-prescription-medicine-provisional-registration-pathway>.

6. Therapeutic Goods Administration. Meeting the eligibility criteria for provisional determination: Australian Government Department of Health and Aged Care; 2018 [updated 11 November 2024. Available from: <https://www.tga.gov.au/resources/guidance/meeting-eligibility-criteria-provisional-determination>.

7. Therapeutic Goods Administration. Applying for provisional registration for a prescription medicine: Australian Government Department of Health and Aged Care; 2018 [updated 8 November 2024. Available from: <https://www.tga.gov.au/resources/guidance/meeting-eligibility-criteria-provisional-determination>.

8. Therapeutic Goods Administration. Applying for provisional registration extension or transition to full registration: Australian Government Department of Health and Aged Care; 2018 [updated 21 January 2021. Available from: <https://www.tga.gov.au/resources/guidance/applying-provisional-registration-extension-or-transition-full-registration>.

9. Perini M., Castiglioni B., Galai E., Trapani D., Genazzani A.A., Miglio G. Differences in the on-label cancer indications of medicinal products between Europe and the USA. Lancet Oncol. 2025;26(2):e103-e10. doi: 10.1016/S1470-2045(24)00434-0.
